# Systematic interpretation of microarray data using experiment annotations

**DOI:** 10.1186/1471-2164-7-319

**Published:** 2006-12-20

**Authors:** Kurt Fellenberg, Christian H Busold, Olaf Witt, Andrea Bauer, Boris Beckmann, Nicole C Hauser, Marcus Frohme, Stefan Winter, Jürgen Dippon, Jörg D Hoheisel

**Affiliations:** 1 Department of Functional Genome Analysis, German Cancer Research Center, PO 101949, D-69009 Heidelberg, Germany; 2 Department of Pediatrics I, University of Göttingen, Robert-Koch-Str. 40, D-37075 Göttingen, Germany; 3 Experimental Pediatric Oncology, German Cancer Research Center, PO 101949, D-69009 Heidelberg, Germany; 4 Fraunhofer IGB, Nobelstr. 12, 70569 Stuttgart, Germany; 5 Institute for Stochastics and Applications, University of Stuttgart, Pfaffenwaldring 57, D-70569 Stuttgart, Germany

## Abstract

**Background:**

Up to now, microarray data are mostly assessed in context with only one or few parameters characterizing the experimental conditions under study. More explicit experiment annotations, however, are highly useful for interpreting microarray data, when available in a statistically accessible format.

**Results:**

We provide means to preprocess these additional data, and to extract relevant traits corresponding to the transcription patterns under study. We found correspondence analysis particularly well-suited for mapping such extracted traits. It visualizes associations both among and between the traits, the hereby annotated experiments, and the genes, revealing how they are all interrelated. Here, we apply our methods to the systematic interpretation of radioactive (single channel) and two-channel data, stemming from model organisms such as yeast and *drosophila *up to complex human cancer samples. Inclusion of technical parameters allows for identification of artifacts and flaws in experimental design.

**Conclusion:**

Biological and clinical traits can act as landmarks in transcription space, systematically mapping the variance of large datasets from the predominant changes down toward intricate details.

## Background

Microarrays completed their first steps involving technical development and extraction of candidate genes by comparing small sets of samples. With the availability of data from public databases, e. g. GEO [1] and ArrayExpress [2], establishment of standards (e. g. MIAME and MAGE, [3]), and more and more hybridizations at hand, attention is now turning to the interpretation of larger datasets spanning many experimental conditions. Various fields of research nowadays have a considerable throughput of microarray experiments. In order to fully interpret those datasets, it is necessary to properly annotate them in all relevant aspects.

However, not only is a sufficient level of detail an issue, but there are also matters of data format [4]. While free text format grants some flexibility for storage, enabling to store protocols from all over the world for example, it complicates statistical access. Even sophisticated text mining methodology does not match the human brain in language interpretation. It often recovers only a small fraction of the information represented in the text that would be available to a human reader. In fact, it is still common practice to read through texts to collect values for variables to account for in subsequent statistical analysis. For large datasets, however, converting textual descriptions into a computer-readable format by human interaction is a tedious task. Alternatively, experiment annotations can be aquired in computer-readable format from the beginning, such that their entire information can be directly subjected to automated analysis [5].

Automated analysis requires that instances of occurance of any annotation are countable. In practice, for large datasets the annotation values need to be identifyable by a computer, i. e. completely specified by fixed terms, using a controlled vocabulary.

In general, microarray data can be viewed as a "genes × experiments" data table. Additional information worthy of being taken into account may be annotated both to the rows or the columns of the table, i. e. the genes and experiments. So far, microarray data annotation mostly means gene annotation. Also, many published methods and software tools are available for interpreting microarray data in the light of gene information, ranging from genomic localization [6,7] over transcription factor binding sites [8] to pathway information [9] or entire GO annotation [10-15], just to name few examples. Comparably little has been done to apply protocol or sample information.

This may, at least in part, result from differences in data structure. For gene annotation, many methods take advantage of the straightforward structure of the Gene Ontology (GO, [16,17]). Most terms apply to nearly all organisms under investigation. In spite of ongoing debates on semantics, the concept prooved successful for data analysis.

On the experiment side, statistical access to such data seems to be a more complex goal. Some parameters, such as size or temperature, are not readily captured by terms but show a continous value range.

Continous ranges need to be discretized (preferably as a first step of the analysis process instead of already annotating inalterable categories) before frequencies of, e. g., small, middle and large individuals can be counted. Further, experiment annotations fundamentally differ from organism to organism. Tumor type makes no sense for yeast research and components of yeast media differ from growth conditions of plants, complicating the development of a common ontology.

To our knowledge, previous publications concerned with statistical analysis of experiment annotations deal with relatively small numbers of traits rather than comprehensive experiment descriptions. Sese *et al. *account for only 16 annotation values [18]. Segal *et al. *extracted 263 values from the literature (rather than from any ontology or microarray database) [19]. These are mainly clinical, i. e. sample-related, lacking information about the experimental protocols involved. Moreover, the clustering algorithms of both publications do not reveal the spread of arrays making up a clinical trait nor do they simultaneously visualize genes, arrays and traits.

In contrast to e. g. the Microarray Gene Expression Data (MGED) Ontology [20], the lists of experiment annotation parameters stored in M-CHiPS (Multi-Conditional Hybridization Intensity Processing System, [21]) are tailored to the specific needs of a particular field of research [5]. Any source of controlled vocabulary may be used. They currently comprise between 547 and 1011 defined values, plus, between 38 and 161 annotations of continuous value ranges, enabling an unprecedented level of detail. Moreover, common to all fields of research, the annotations cover technical parameters of array, RNA preparation, labelling, hybridization, washing and signal detection in order to pin down artifacts.

Not all of the annotated traits correspond to the signal patterns observed in an experimental context under study and it is not known in advance which of them do. We provide means to preprocess these annotations and extract traits that correlate with transcription. Those are visualized by correspondence analysis (CA) [22,23]. CA has been shown to reveal intricate details not visible with hierarchical clustering [23]. Like other projection methods, it represents the columns of our "genes × experiments" data table as vectors in a high-dimensional space spanned by its rows (or vice versa). We will refer to this space as 'transcription space'. Any possible status of the transcriptome (e. g. of one of our samples) occupies a particular position in it. For visual inspection, the data points are projected onto a two-dimensional map that accounts for the main variance in the data.

Whereas both experiment annotations [5] and CA of microarray data [22,23] have been reported earlier, the two approaches remained unconnected yet. By representing the experiment annotations in transcription space, we enable CA to account for associations both among and between single genes, experiments and relevant experiment annotations in a single plot. Moreover, each trait can be judged according to the spread of hybridizations annotated thereof. One can recognize it as being either locally confined or spanning a wider area in transcription space and genes can be identified as being associated to it. In mapping the transcription space with well-known tags, the experiment annotations allow for a "guided tour" through the interpretable variance of the transcription data. For systematic interpretation a top-down and a bottom-up approach are presented. Moreover, we found experiment annotations particularly valuable in detecting artifacts as well as flaws in experimental design. To demonstrate the general applicability of our methods, we show data examples aquired with single and two-channel platforms, stemming from yeast, fruit fly and human cancer.

## Results

**Figure 1:**
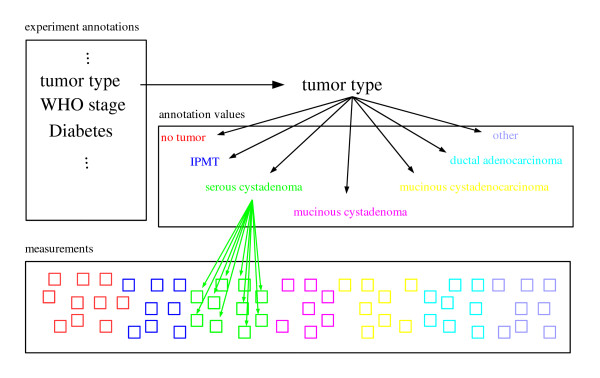
**Experiment annotations, annotation values, and measurements**. A set of experiment annotations (left upper box) is assessed describing an experimental context, here a microarray study of pancreas cancer samples. Each experiment annotation (e. g. tumor type) can take several annotation values (right upper box). Each of these annotation values (e. g. serous cystadenoma) annotates a set of microarray measurements. Each measurement is affiliated to exactly one annotation value per experiment annotation. In this manner, each experiment annotation represents a possible grouping of the measurements, with each experiment annotation value representing a distinct group. If this grouping corresponds to the transcription patterns observed, the experiment annotation is relevant for the experimental context under study.

Each dataset was normalized and filtered (see Methods and Additional file 1). It may be regarded as a table, each row representing a gene (that meets the filter criteria), each column standing for a transcription measurement. In the following, we refer to any measured atomic set of values of a hybridization, e. g., the Cy5 channel for half of the spots on an array with each gene spotted in duplicate, as "measurement". An experimental condition is recorded by multiple measurements, also involving repeated sampling, labelling, and hybridization. Additional parameters or variables provide further information about the measurements. We will refer to these variables (or *factors*) as "experiment annotations", to the values (*levels*) taken by these as "annotation values". Figure 1 illustrates these terms. In transcription space, each annotation value is represented as the centroid (weighted average) of the according experimental conditions.

### Correspondence analysis

Basis for visualization is a data table, rows representing the genes, columns the experimental conditions or annotation values (each summarizing a set of conditions). The information content of such a table can be judged by its total inertia which is computed by deviding the χ^2 ^statistic of the table by its grand total. High associations between rows and columns are reflected by a large inertias (as opposed to homogenous table entries whose small differences could occur due to chance alone). In this sense, a "differential" gene, i.e. a gene deviating from its "normal expression", in a particular column, shows a large contribution to the total inertia of the table. The larger the deviation from its expected value, the farther the gene will be located from the plot center. It will lie in the same direction as the associated column (condition or annotation value) in case of positive association, or in the opposite direction if it is particularly downregulated in this column. If several transcriptionally related conditions that are also annotated by the same annotation value are combined into one column representing this annotation value, it will be located in the center of gravity of the combined conditions, with all associated genes being located in the same direction.

### Transcription patterns integrated with clinical data (overview)

Before focusing on biologically relevant parameters, the variance should be critically assessed for potentially confounding effects that may distort further analysis or that may even abolish comparability of the data within larger datasets. The supplementary material (Additional file 1) provides two data examples demonstrating that experiment annotations are capable of detecting analysis-interfering artifacts and pitfalls in experimental design.

The following dataset assessing pancreas carcinoma along with healthy tissue samples was selected to demonstrate the methods' applicability for navigation through a large number of biologically relevant parameters that correlate with transcripton (Table 3 in the Additional file 1).

Investigating the data with no further hypothesis by exploratory data analysis (data mining), it appears desirable to visualize all parameters in a crude overview before breaking down the interpretable variance into details (top-down approach).

But not all annotation values should be taken at face value. Some do not carry considerable amounts of information in terms of transcription behaviour. We assess this by computing their inertia contributions. The inertia, computed as the χ^2 ^statistic devided by the grand total of the data table, is a means of assessing the variance or information content of a data table. Here, each table column contains the (prototype) transcription profile of a particular experiment annotation value, contributing a certain share to the total inertia of the table. The discretized annotation values are filtered according to the variance they contribute in the context of all values of all annotations (Tab. 5 in Additional file 1).

However, this criterion alone is not sufficient for filtering relevant experiment values. Each of the annotation values is a centroid of all experimental conditions annotated by this value. These conditions may cluster densely around their centroid, well-separated from all other conditions, or they may show inhomogenous transcription, overlapping with conditions annotated by another value. To assess if an annotation value annotates a distinct cluster of conditions or not, we compute the Silhouette value (SV, [24]). Let there be an experimental annotation **A **taking values *i*∈**A**. One SV per annotation value *i *and measurement *j *is computed as *s*_*ij *_= (*b*_*ij *_- *a*_*ij*_)/*max*(*a*_*ij*_, *b*_*ij*_), where *a*_*ij *_is the average distance of annotated measurement *j *to all other measurements annotated with *i *and *b*_*ij *_is the minimum of average distances of measurement *j *to all measurements not annotated with *i*. Here, the Silhouette scores were computed on the basis of the χ^2 ^distances.

A SV close to one will result for measurements well-separated from the measurements of neighboring clusters (composed of measurements annotated with annotation values other than *i*). A score around zero means that the measurement could be assigned to another annotation value, as well. A score close to minus one denotes that the object is most likely misclassified, i. e. transcriptionally affiliated to another but the annotated annotation value. The average SV of all measurements annotated with a particular annotation value *i *is used as a second criterion for filtering the annotation values (Tabs. 4 and 5 in the Additional file 1).

**Figure 2:**
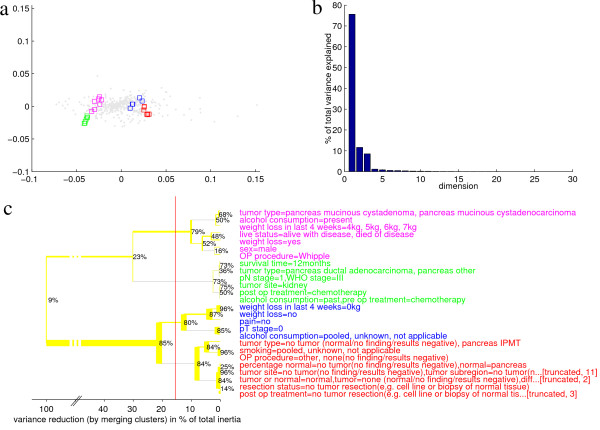
**Overview of the pancreas cancer data**. Human biopsies are characterized in terms of 26 (out of 93) experiment annotation values that have been selected for reproducibly corresponding to major variances in transcription. These traits have been subdevided into four different clusters (red, blue, pink, and green) by cutting the hierarchical clustering tree (panel c) at less than 20% of their total variance. Thickness of lines in the clustering tree corresponds to numbers of hybridizations annotated with at least one of the traits of the according cluster. The thickness of the horizontal yellow lines corresponds to the number of measurements posessing the listed trait, or, in case of a feature-cluster, at least one of the comprised traits, but none of the traits of the cluster to merge with next. A grey line stands for the empty set, indicating that, in terms of annotated measurements e. g. of the green cluster, this cluster is completely included in the cluster to merge with. The thickness of the vertical line indicates the cardinality of the intersection (number of measurements having at least one trait out of either cluster). Whereas the line thickness is proportional to the number of measurements relative to the total number of measurements in the dataset, the percentages written next to the vertical lines denote the cardinality of an intersection relative to the cardinality of the union of the particular two clusters to merge, only. The annotation values are also shown by CA (panel a), genes being plotted as grey dots, traits as boxes color-coded as above. The plot reveals that the difference between the first two and the second two clusters corresponds to many differential genes and makes up to 75% of the total variance among the traits (panel b).

Among all 93 values taken by any annotation of the data after discretization (Table 5 in the Additional file 1), Fig. 2 plots the 26 filtered out for showing inertia contributions above 1% and positive SV, i. e. considerable variance of the values and clustering of measurements annotated thereof. They are shown both by CA (Fig. 2a, variance explained by the principal axes shown in Fig. 2)b as well as by hierarchical clustering (Fig. 2)c.

Two transcriptionally relevant features A and B cluster together, if A and B are similar in terms of transcription behaviour of the samples having these traits. The sets of measurements annotated by A and B may overlap by many measurements showing both features. On the other hand, although neighbouring in transcription space, they may also be completely disjoint (leading to a differing interpretation). Thus, apart from the similarity in transcription, the overlap (percentage of intersecting measurements) can be read out for neighbouring features in Fig. 2c.

Features annotating identical sets of measurements (e. g. pN stage 1 and WHO stage III) and those discretized into one category (e. g. normal tissues and IPMT) have been combined into one. In Fig. 2c, the merging of many features potentially yields longer rows. For the truncated rows, the number in square brackets refers to the rank in Table 5 of Additional file 1.

The hierarchical clustering has been performed by merging two clusters, if the variance-reduction introduced by combining this particular pair is minimal among all possible pairs. The variance-reduction for each merge can be read out from the scale at the bottom of Fig. 2c. Here, in order to obtain a broad overview over the transcription space, the hierarchical tree is cut arbitrarily at the level of four resulting clusters (red vertical line), such that all but one more general cluster comprise a characteristic tumor type. The more general (blue) cluster would actually contain the annotation value 'tumor type = pancreas serous cystadenoma' (data not shown), which is an inhomogenous class in terms of transcription profiles and was therefore filtered out for negative SV.

These clusters can be interpreted in terms of their annotated features. Ordered by increasing malignancy, the first cluster (red, "normal") comprises normal tissue as well as intraductal papillary mucinous tumor (IPMT, in our case all benign) samples. The second one (blue, "general" or "serous") containing the serous and other tumor samples, comprises more general features such as no pain, no weight loss, and pT stage zero (no primary tumor identified, [25]) that apply to a wider area in transcription space. The third cluster (pink, "mucinous") comprises the mucinous tumor types along with present alcohol consumption, diabetes, weight loss and moderate pain. The only sample annotated with 'severe pain' was of uncertain pathology. It is characteristic for pancreatic cancer, that patients sense no greater pain before a late stage, in which, due to metastazation, the cancer is inoperable in most cases and is thus not among the biopsies studied here. The most malignant type of pancreas cancer contained by the data (green, "ductal") is characterized by short-term survival, pN stage 1, WHO stage III, metastases ('tumor site = kidney'), pre- and post-operational chemotherapy, and, interestingly, past alcohol consumption.

Fig. 2a shows a CA visualizing 87.2% (sum of first two dimensions in Fig. 2b) of the total inertia of the filtered annotation values. These are color-coded according to above clusters. As in Fig. 2c, the variance among and within the clusters is visualized. Also, CA (Fig. 2a) confirms that the predominant variance is between the two malignant clusters (green and pink) on one side and the two more benign ones (blue and red) on the other. Extending what is visible from Fig. 2c, CA also shows the genes responsible for this. It tends to display genes (grey dots) associated to, i. e. upregulated under a particular condition on a line from the plot center to this condition, the stronger the association, the larger the distance to the center. The majority of the differential genes (i. e. those with greater distance to the plot center) correspond to this difference. Their transcripts are most abundant either both in the first two or both in the second two clusters. A much lower number of genes discriminates between the two malignant and/or between the two benign clusters.

**Figure 3:**
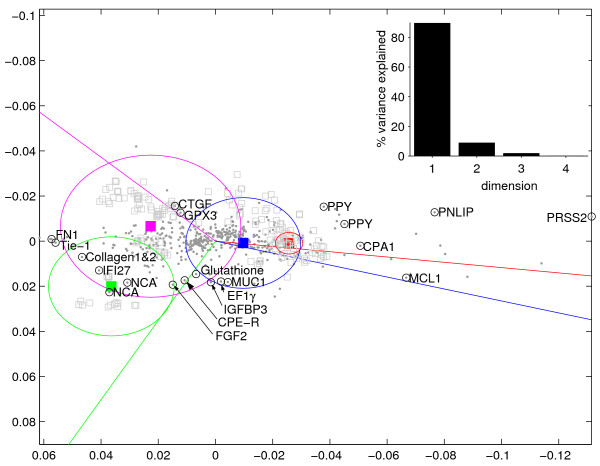
**Trait-cluster ranges**. The cluster centroids of the experiment annotation values of Fig. 2 have been projected by CA, the first two principal axes explaining almost the entire variance among these (upper right corner). Genes are depicted as grey dots, hybridization measurements (plotted without mass) as grey empty boxes, cluster centroids as filled boxes, color coded as in Fig. 2 Around each centroid, a circle incloses 80% of the measurements annotated with at least one of the traits belonging to the particular cluster. Lines to the cluster centroids in standard coordinates [23] indicate the direction of highest association with a certain cluster for the genes. Some of these are encirceled in black, tagged by a gene name and further referred to in the text.

In order to accurately afflilate associated genes to the above clusters, the individual traits have been combined to one representative transcription profile per cluster (cluster-centroid). Fig. 3 shows a CA projecting the variance of the genes according to these cluster-centroids, explaining 98.3% of the inter-cluster variance (upper right corner). As already visible in Fig. 2c, the four clusters overlap to differing amounts in terms of the sets of annotated measurements. Their individual ranges are scetched by circles comprising 80% of all measurements (grey squares) annotated with at least one of the traits in the cluster. The center of the circle is in the location of the transcription profile most representative for the particular combination of traits. The least malignant cluster 1 (red) including the control tissues, for example, has the smallest range, representing by and large a subset of cluster 2 (blue) while not intersecting with the cluster 4 (green) of high malignancy.

Many genes associated to cluster 1 (red) and 2 (blue) are indicative of normal, differentiated and functional pancreatic tissue (PRSS2, PNLIP, MCL1, CPA1, PPY), encoding for proteins required for food digestion. Exclusively associated to cluster three (magenta, mucinous tumors) are the connective tissue growth factor (CTGF) which will be discussed in context of present alcohol consumption as well as the glutathione peroxidase 3 (GPX3) which was reported to be overexpressed in ovarian cancer [26]. On the left, we find genes associated with both cluster 3 and 4 (FN1, Tie-1, Collagen, IFI27, NCA), indicating e. g. proliferation of epithelial and interstitial connective tissue. Further discussion and literature are provided in Additional file 1.

Exclusively associated to the fourth cluster (green, highest malignancy) are genes applicable to discriminating the highly agressive tumors from the mucinous, such as the fibroblast growth factor 2 (FGF2, consistent with [27]), the *Clostridium perfringens *enterotoxin receptor (CPE-R) which was discussed in context of prostate cancer [28], and Glutathione [29]. Also applicable to discriminating the highly agressive tumors from the mucinous are mucin 1 (MUC1), which is in agreement with [30], as well as two more genes described as follows. Increasing evidence has accumulated in support of the hypothesis that growth hormone (GH) and insulin-like growth factors (IGFs) play a role in carcinogenesis. Insulin like growth factor binding protein 3 (IGFBP3) is upregulated in pancreatic endocrine tumors and its overexpression is significantly more common in metastatic disease [31]. High expression of IGFBP3 has been associated with invasiveness and poor prognosis in other cancer types [32]. GH receptor antagonist treatment decreased colon carcinoma growth in nude mice, associated with reduction in circulating IGFBP3 levels [33]. Elongation factor 1*γ *(EF1*γ*) is overexpressed in esophageal cancer with severe lymph node metastasis and far advanced stages of the disease compared with non-overexpressing cases [34]. In summary, genes affiliated to cluster four are known to be associated with metastasis, advanced stage disease and poor prognosis of pancreatic and other cancers.

After assessing the variance between the trait-clusters, one can go into more detail by analyzing each cluster alone, assessing the variance within. This strategy can be recursively taken to increasingly smaller variances until inspecting differences between single traits. Discussing the entire variance of the pancreas dataset is beyond the scope of this paper. Figs. 6 and 7 in the Additional file 1 show the analysis of cluster four, focusing on the most malign samples.

### Picking aspects of interest (details)

**Figure 4:**
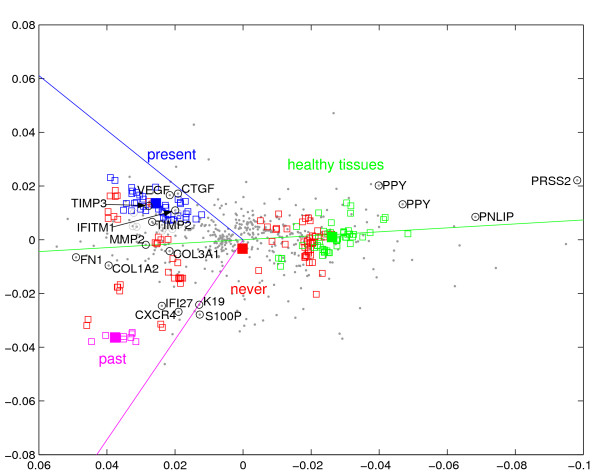
**Alcohol consumption**. The annotation values of experiment annotation alcohol consumption' (solid boxes) have been projected by CA. Elements are drawn as in Fig. 3. Unlike in Fig. 3, measurements (empty boxes) are color-coded. Each measurement corresponds to only one annotation value, because the map is limited to one experiment annotation. We reversed the direction of the abscissa to maintain the orientation of tumors versus healthy tissues of the previous figures. We changed the color-code, however, to acknoledge the fact that present alcohol consumption alone does not represent the entire pink trait cluster of Fig. 2, for example.

In a bottom-up (agglomerative) approach, the experiment annotations may serve to first pick aspects of particular interest, investigating the data aspect by aspect. In the extreme, a single annotation or only a subset of its values can be projected, before visualizing certain aspects together to assess their interaction. Knowing from Tab. 3 (Additional file 1) which parameters correlate to transcription, one can select one or several of special interest. Fig. 4 projects the values taken by the annotation 'alcohol consumption' (Tab. 4 in the Additional file 1). Its right half ('healthy tissues') displays genes already discussed for the overview such as PPY, PNLIP and PRSS2, which are expressed by healthy pancreatic cells for food digestion and which are downregulated upon alcohol consumption (both past and present). On the opposite side along the green line, genes are located which are upregulated with both past and present alcohol consumption: Fibronectin (FN1), and collagens Type I (COL1A2) and III (COL3A1) have already been discussed above in context of the strong desmoplastic reaction of pancreatic cancer. Matrix Metalloproteinase 2 (MMP2) has been found to be expressed in pancreatic cancers and has been positively correlated with metastasis [35,36]. Furthermore, MMP2 has been found to be a diagnositc marker for pancreatic carcinoma in pancreatic juice [37]. In summary, the geneset negatively or positively associated to alcohol consumption in general characterizes healthy pancreatic tissue on one hand and the dense connective tissue reaction of pancreatic cancer involving fibronectin and collagens type I and III on the other.

The difference between present and past alchohol consumption is shown statistically significant and extensively discussed in terms of associated genes in Additional file 1. In summary, genes associated with past alcohol consumption have been linked to physiological processes associated with increased risk for malignant transformation, pancreatic cancer cell proliferation, survival, invasion, metastasis, and impaired cell differentiation (K19, IFI27, S100P, CXCR4). In contrast to past alcohol consumption, present alcohol intake appears associated with the expression of immediate response genes to tissue damage, repair and remodeling, inflammatory and stress response (IFITM1, CRHBP, TIMP 2 and 3, DUSP1, CTGF).

## Discussion

Whereas there is a wealth of methods published to analyze microarray data together with gene annotation, little has been done to integrate experiment annotation related to experimental protocols and sample description. General titles about ontology-driven analysis of microarray data often obscure that a publication is concerned solely with gene annotations. That does not mean that experiment annotations are regarded dispensable or not being worked on. There are projects under way to explicitly capture sample and experiment descriptions in unified ways. Also, there are first attemtps to statistically analyze particular traits together with the transcription data. But the two approaches have remained unconnected yet.

We use large hierarchically ordered lists of factors in a way that renders all the data readily available for statistical access. Rather than using only one framework for all fields of microarray research, these lists satisfy the specific requirements of each field, allowing for arbitrary levels of detail. We take into account also technical parameters to identify artifacts as well as flaws in experimental design. In providing means to statistically analyze explicit experiment descriptions covering all aspects that may be relevant, our approach links between sophisticated and holistic, yet complex, incomplete and therefore not readily statistically accessible standardization on one hand, and statistical analysis of few traits manually extracted from the literature on the other.

When processing detailed experiment descriptions, not all annotated traits correspond to transcription in a particular experimental context. They need to be preprocessed and filtered. Afterwards, it is advisable to first consider technical artifacts before proceeding to biological variations that may well comprise smaller variance in some cases. After dealing with the technical variance feeding into the design of follow-up experiments, or simply resulting in the exclusion of hybridizations from further analysis, biological variabilities can be visualized. Apart from the advantages listed in ref. [23], CA is particularly convenient for the integration of characteristic traits common to more than one experiment because it works on the χ^2 ^distance. This is the only one of its kind possessing a property called the 'principle of distributional equivalence' [38]: If column profiles are identical or similar, the corresponding columns can be merged by summation with no or little change to the positions of the rows. In our context, this guarantees the stability in distances between genes, when similar experimental conditions are merged into a representative trait common to all of them.

**Figure 5:**
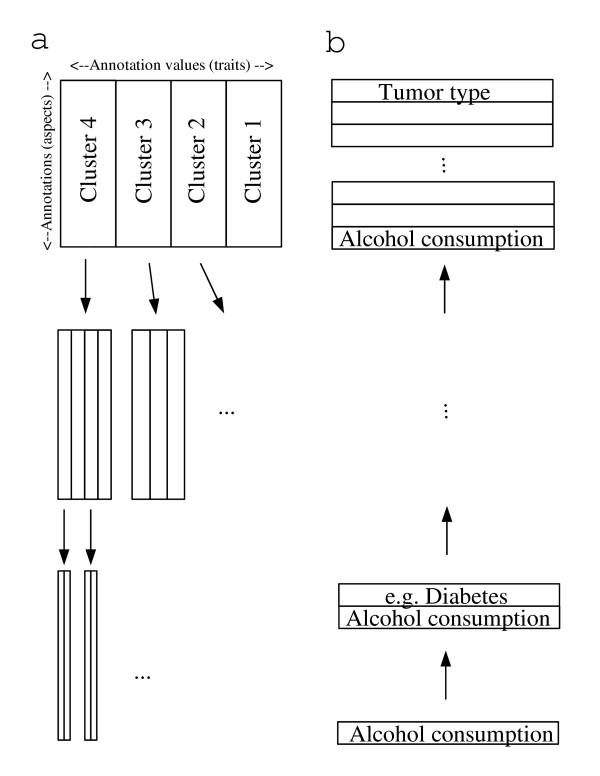
**Systematic interpretation**. For systematic interpretation of large datasets, the comprised variance can be divisively split up in a "top-down" "by-trait" assessment shown in panel a: After regarding the variance between a small number of trait clusters (Figs. 2 and 3), the variance within each cluster is analyzed separately in the same way (Figs. 6 and 7 in the Additional file 1) until analyses consist of single traits. Thus, the top-down approach proceeds from the predominant variance to more subtle changes, answering the question which traits are different and which similar transcriptionally. In contrast, an agglomerative "bottom-up" approach will focus on few traits initially (panel b). These may stem from a single annotation (aspect, parameter) of special interest (e. g. alcohol consumption, Fig. 4). In further steps, the most interesting annotation (not necessarily representing large variance) is combined with other aspects to visualize their interaction.

Thus visualized, experiment annotations can be employed as landmarks in transcription space. They can be used to map crude overviews down to intricate details (which may need statistical validation), allowing for a systematic interpretation of the entire variance of large datasets (Fig. 5). There is no way of discussing the entire interpretable variance of the pancreas data within this paper. Both a top-down and a bottom-up approach are sketched in example by their first step. Not all of the biological variance may be of interest to a researcher. In a bottom-up (agglomerative) approach, the experiment annotations may serve to first pick aspects of particular interest, investigating the data aspect by aspect (Fig. 5b). In extreme, a single annotation (Fig. 4) or only a subset of its values can be projected, before adding more annotations to study their interaction.

For explorative data analysis, however, it is more convenient to plot an overall overview as a first step before devisively splitting up the variance (Fig. 5a). Figure 2 shows all traits meeting our filter-constraints, both projected by CA (Fig. 2a) and hierarchically clustered (Fig. 2c). By means of the latter, more general clusters can be identified. The single traits, by meeting the filter-criteria, stem from tightly clustered experimental conditions and correspond to relatively small, well-defined areas in transcription space. In contrast, the more general clusters show different ranges in transcription space (Fig. 3).

Another consequence of reducing the accounted variance to a small number of cluster-centroids is the enhanced projection quality. The two dimensions shown in Fig. 2a explain only 87% (Fig. 2b) of the variability among the single traits. The same applies to the variability of the genes in respect of these traits. In practice, that means that many genes cannot be accurately characterized in two dimensions (but stick out of the paper plane, mostly in the third dimension which explains 8.6%, Fig. 2).

In general, n objects can be projected onto an n-1 dimensional (hyper-) plane such that all their distances are preserved. As a consequence of reducing to three or four data points in gene-dimensional space, the two-dimensional CA plot perfectly or almost perfectly explains the complete remaining variance (98.3% in Fig. 3), respectively. Differential genes can be accurately affiliated to the feature clusters, reflecting the behaviour of genes in an 'average' measurement showing the traits of one of the four clusters.

Thus, few large areas in transcription space are characterized in terms of associated experiment annotation values as well as in terms of corresponding genes. The process may be iterated, investigating any of these areas (i. e. feature clusters) alone and recursively taken to subtle transcription patterns as a "guided tour" through the interpret able variance.

## Conclusion

Currently, data interpretation represents the main bottleneck in transcriptional profiling experiments on microarrays [39]. Experiment annotations provide a means to systematically interpret microarray data. Represented as prototype (i. e. typical) profiles in transcription space, the experiment annotation traits function as tags for organizing an overwhelming amount of information for interpretation, breaking it down into digestible pieces that can be understood and catalogued. Yet more variables, such as promotor sequences and protein levels could be integrated by CA, either as done so far, i. e. by applicative arrangement in a two-way table, or by multiple or joint correspondence analysis.

## Methods

### Experimental methods and preprocessing of transcription data

Sampling, labeling by reverse transcription, and hybridization were performed as described [40-47]. Detailed experiment annotations in statistically accessible format (controlled vocabulary) can be obtained for the yeast data from the Eurofan II, B2 web page [48] for all other data from our web page [49]. Normalization of single-channel data (involving radioactive label) was carried out according to ref. [23]. The method is based on a log-linear normalization described in ref. [50] performing better than or equally to lowess normalization [51]. In contrast, for the two-channel (fluorescent label) platform, involving a control-channel refering to a reference condition hybridized on all chips, each non-control channel was normalized with respect to the control-channel of the particular hybridization [52].

We select genes showing significant absolute expression level in at least one of the conditions under study, substantial change relative to the control condition in at least one of the other conditions, and reliable reproducibility in the separation from the control condition in at least one of the other conditions [50]. Details can be obtained from Additional file 1.

### Correspondence analysis

We provide here a concise summary of the technique (see refs. [53] and [38] for details). A projection method much like principle components analysis (PCA, [54,55]), CA takes as input a matrix of genes × experiments and aims at projecting these data into a subspace of low dimensionality, e. g. a plane. In contrast to PCA, CA embeds both rows (genes) and columns (experiments) of the matrix in the same space. Let *I *genes and *J *experiments form *I *× *J *matrix **N **with elements *n*_*ij*_. Let *n*_*i*+ _and *n*_+*j *_denote the sum of the *i*th row and *j*th column, respectively. Let *n*_++ _be the grand total of **N**. The mass of the *j*th column is defined as *c*_*j *_= *n*_+*j*_/*n*_++_, the mass of the ith row is *r*_*i *_= *n*_*i*+_/*n*_++_. We compute the correspondence matrix **P **with elements *p*_*ij *_= *n*_*ij*_/*n*_++_, and matrix **S **with elements *s*_*ij *_= (*p*_*ij *_- *r*_*i*_*c*_*j*_)/ricj
 MathType@MTEF@5@5@+=feaafiart1ev1aaatCvAUfKttLearuWrP9MDH5MBPbIqV92AaeXatLxBI9gBaebbnrfifHhDYfgasaacH8akY=wiFfYdH8Gipec8Eeeu0xXdbba9frFj0=OqFfea0dXdd9vqai=hGuQ8kuc9pgc9s8qqaq=dirpe0xb9q8qiLsFr0=vr0=vr0dc8meaabaqaciaacaGaaeqabaqabeGadaaakeaadaGcaaqaaiabdkhaYnaaBaaaleaacqWGPbqAaeqaaOGaem4yam2aaSbaaSqaaiabdQgaQbqabaaabeaaaaa@3292@. **S **is subjected to singular value decomposition [56]. It is decomposed into the product of three matrices: **S **= *U*Λ*V*^*T*^. Λ is a diagonal matrix, its diagonal elements *λ*_*k *_being the singular values of **S**. For the projection, coordinates of gene *i *are computed as *f*_*ik *_= *λ*_*k*_*u*_*ik*_/ri
 MathType@MTEF@5@5@+=feaafiart1ev1aaatCvAUfKttLearuWrP9MDH5MBPbIqV92AaeXatLxBI9gBaebbnrfifHhDYfgasaacH8akY=wiFfYdH8Gipec8Eeeu0xXdbba9frFj0=OqFfea0dXdd9vqai=hGuQ8kuc9pgc9s8qqaq=dirpe0xb9q8qiLsFr0=vr0=vr0dc8meaabaqaciaacaGaaeqabaqabeGadaaakeaadaGcaaqaaiabdkhaYnaaBaaaleaacqWGPbqAaeqaaaqabaaaaa@2FB0@, of hybridization *j *as *g*_*jk *_= *λ*_*k*_*v*_*jk*_/cj
 MathType@MTEF@5@5@+=feaafiart1ev1aaatCvAUfKttLearuWrP9MDH5MBPbIqV92AaeXatLxBI9gBaebbnrfifHhDYfgasaacH8akY=wiFfYdH8Gipec8Eeeu0xXdbba9frFj0=OqFfea0dXdd9vqai=hGuQ8kuc9pgc9s8qqaq=dirpe0xb9q8qiLsFr0=vr0=vr0dc8meaabaqaciaacaGaaeqabaqabeGadaaakeaadaGcaaqaaiabdogaJnaaBaaaleaacqWGQbGAaeqaaaqabaaaaa@2F94@, for *k *= 1,...,*J*. These coordinates are called principal coordinates.

So called standard coordinates for the columns (experimental conditions) of the data matrix can help to identify associated genes. They are computed as *v*_*jk*_/cj
 MathType@MTEF@5@5@+=feaafiart1ev1aaatCvAUfKttLearuWrP9MDH5MBPbIqV92AaeXatLxBI9gBaebbnrfifHhDYfgasaacH8akY=wiFfYdH8Gipec8Eeeu0xXdbba9frFj0=OqFfea0dXdd9vqai=hGuQ8kuc9pgc9s8qqaq=dirpe0xb9q8qiLsFr0=vr0=vr0dc8meaabaqaciaacaGaaeqabaqabeGadaaakeaadaGcaaqaaiabdogaJnaaBaaaleaacqWGQbGAaeqaaaqabaaaaa@2F94@. In practice, plotting them would shrink all remaining points to a tiny area. We therefore draw a line from the center of the plot to each standard coordinate instead of plotting the standard coordinates themselves. We depict the first two dimenstions (*k *= 1, 2) of the projection space. The loss of information associated with this dimension reduction can be computed as fraction of the total inertia ∑kλk2
 MathType@MTEF@5@5@+=feaafiart1ev1aaatCvAUfKttLearuWrP9MDH5MBPbIqV92AaeXatLxBI9gBaebbnrfifHhDYfgasaacH8akY=wiFfYdH8Gipec8Eeeu0xXdbba9frFj0=OqFfea0dXdd9vqai=hGuQ8kuc9pgc9s8qqaq=dirpe0xb9q8qiLsFr0=vr0=vr0dc8meaabaqaciaacaGaaeqabaqabeGadaaakeaadaaeqaqaaGGaciab=T7aSnaaDaaaleaacqWGRbWAaeaacqaIYaGmaaaabaGaem4AaSgabeqdcqGHris5aaaa@341C@ explained by *k *= 1, 2, i. e. (λ12+λ22)/∑kλk2
 MathType@MTEF@5@5@+=feaafiart1ev1aaatCvAUfKttLearuWrP9MDH5MBPbIqV92AaeXatLxBI9gBaebbnrfifHhDYfgasaacH8akY=wiFfYdH8Gipec8Eeeu0xXdbba9frFj0=OqFfea0dXdd9vqai=hGuQ8kuc9pgc9s8qqaq=dirpe0xb9q8qiLsFr0=vr0=vr0dc8meaabaqaciaacaGaaeqabaqabeGadaaakeaadaqadaqaaGGaciab=T7aSnaaDaaaleaaieaacqGFXaqmaeaacqaIYaGmaaGccqGHRaWkcqWF7oaBdaqhaaWcbaGaeGOmaidabaGaeGOmaidaaaGccaGLOaGaayzkaaGaei4la8YaaabeaeaacqWF7oaBdaqhaaWcbaGaem4AaSgabaGaeGOmaidaaaqaaiabdUgaRbqab0GaeyyeIuoaaaa@3EFE@. CA offers the opportunity to embed rows or columns "without mass", i. e. without being taken into account for computing the projection space. Let the matrix **N **determine the projection and let N* of elements nij′*
 MathType@MTEF@5@5@+=feaafiart1ev1aaatCvAUfKttLearuWrP9MDH5MBPbIqV92AaeXatLxBI9gBaebbnrfifHhDYfgasaacH8akY=wiFfYdH8Gipec8Eeeu0xXdbba9frFj0=OqFfea0dXdd9vqai=hGuQ8kuc9pgc9s8qqaq=dirpe0xb9q8qiLsFr0=vr0=vr0dc8meaabaqaciaacaGaaeqabaqabeGadaaakeaacqWGUbGBdaqhaaWcbaGaemyAaKMafmOAaOMbauaaaeaacqGGQaGkaaaaaa@31DE@, contain columns to be plotted without mass. **N **is submitted to correspondence analysis. Let P* have elements pij′∗=nij′∗/n++∗
 MathType@MTEF@5@5@+=feaafiart1ev1aaatCvAUfKttLearuWrP9MDH5MBPbIqV92AaeXatLxBI9gBaebbnrfifHhDYfgasaacH8akY=wiFfYdH8Gipec8Eeeu0xXdbba9frFj0=OqFfea0dXdd9vqai=hGuQ8kuc9pgc9s8qqaq=dirpe0xb9q8qiLsFr0=vr0=vr0dc8meaabaqaciaacaGaaeqabaqabeGadaaakeaacqWGWbaCdaqhaaWcbaGaemyAaKMafmOAaOMbauaaaeaacqGHxiIkaaGccqGH9aqpcqWGUbGBdaqhaaWcbaGaemyAaKMafmOAaOMbauaaaeaacqGHxiIkaaGccqGGVaWlcqWGUbGBdaqhaaWcbaGaey4kaSIaey4kaScabaGaey4fIOcaaaaa@3D7F@. The principal coordinates for the supplementary columns from correspondence matrix P* are then computed as

gj′k∗=1∑ipij′∗∑ipij′∗fikλk.
 MathType@MTEF@5@5@+=feaafiart1ev1aaatCvAUfKttLearuWrP9MDH5MBPbIqV92AaeXatLxBI9gBaebbnrfifHhDYfgasaacH8akY=wiFfYdH8Gipec8Eeeu0xXdbba9frFj0=OqFfea0dXdd9vqai=hGuQ8kuc9pgc9s8qqaq=dirpe0xb9q8qiLsFr0=vr0=vr0dc8meaabaqaciaacaGaaeqabaqabeGadaaakeaacqWGNbWzdaqhaaWcbaGafmOAaOMbauaacqWGRbWAaeaacqGHxiIkaaGccqGH9aqpdaWcaaqaaiabigdaXaqaamaaqafabaGaemiCaa3aa0baaSqaaiabdMgaPjqbdQgaQzaafaaabaGaey4fIOcaaaqaaiabdMgaPbqab0GaeyyeIuoaaaGcdaaeqbqaamaalaaabaGaemiCaa3aa0baaSqaaiabdMgaPjqbdQgaQzaafaaabaGaey4fIOcaaOGaemOzay2aaSbaaSqaaiabdMgaPjabdUgaRbqabaaakeaaiiGacqWF7oaBdaWgaaWcbaGaem4AaSgabeaaaaaabaGaemyAaKgabeqdcqGHris5aOGaeiOla4caaa@4E0C@

### Representation of experiment annotation data in transcription space

For each experiment annotation, its values correspond to disjoint sets of measurements. Fig. 4 shows an example. All measurements sharing an annotation value are depicted as empty boxes of the same colour. Measurements have been repeatedly performed for each particular experimental condition (in context of Fig. 4, that means for a particular patient). Robust transcription profiles representing the experimental condition can be obtained by the gene-wise median of its measurements [23]. Here, among all measurements annotated by a particular annotation value, we compute the gene-wise median for those belonging to the same condition. The data table for a particular annotation therefore contains one column for each experimental condition that is uniformly annotated by the particular annotation. Thus, the weight of a particular annotation value corresponds more to the number of hereby annotated conditions than to the number of repeatedly performed hybridizations that may vary among different conditions.

The χ^2 ^distance possesses the property of distributional equivalence [38]. For a CA in this context, that means that table columns (experimental conditions) showing similar transcription profiles can be merged into one representative profile without changing even the location of the rows (genes), simply by row-wise adding the according columns. Here we add up all the columns annotated by the same annotation value to one representative transcription profile. Thus, in transcription space, each annotation value is represented as the centroid of the according experimental conditions (filled boxes in Fig. 4).

In CA, we compute the principal axes according to the annotation values, projecting the actual measurements without mass. To further simplify interpretation, annotation values showing similar representative transcription profiles can be agglomeratively combined by hierarchical clustering. In the approach described by ref. [57], two clusters are combined, if the variance-reduction introduced by combining this particular pair is minimal among all pairs. Again, the combination is carried out by adding the profiles.

### Preprocessing of experiment annotations

Much like the transcription data, the experiment annotations should not be taken at face value. Many of them may not relate to transcription. Other annotations may correlate with the experimental processes under study but show a continous value range that needs to be discretized into meaningful groups of values. Discretization into meaningful groups of values is by no means trivial. Depending on the experimental context, different groupings of the same value range may be most informative, which is the reason for preprocessing the transcription data 'on the fly' beforehand, instead of using predefined intervalls. Group centers or borders of informative groupings may appear equidistant in different scales (e. g. linear or log-transformed) for different annotations or may be not regularly spaced at all. From a biological point of view, it can even occur that single ranges are not uniformly continuous. In adaption processes, e. g. responses to transient environmental changes or in cyclic processes such as the cell-cycle, the transcriptome of cells tends to converge to the initial point after a while such that the lowest and the highest values of e. g. a time variable may well annotate a common cluster of measurements with the middle range corresponding to other transcription states.

Also, high numbers of values taken by nominal annotations may raise the need to group values without any obvious measure of similarity. In other cases, the order of enumeration type values is a matter of debate. Classifying a parameter as nominal or ordinal already means to take into account external biological knowledge about the annotation to discretize. Human interaction is necessary.

In most cases, the biologist provides some good idea of similarity among the values, e. g. of similarity among certain tumor types. In this case, the most informative grouping will maximize the correlation of similarity/dissimilarity among these groups of annotation values on one hand and the similarity/dissimilarity of the transcription profiles of the corresponding measurements on the other. By clustering the transcription profiles of the annotation values by hierarchical clustering we let the biologist decide about a suitable level for cutting the tree, taking into account whatever order or similarity information suitable for the individual parameter.

This is done separately for each annotation that takes more values than suitable for simplicity and clarity of visualization, regardless of its range. Here, for each dataset, all annotations taking more than four values were discretized (see Additional file 1).

Afterwards, the discretized annotation values have been ranked and/or filtered according to the variance they contribute either in the context of the values of one annotation or of all annotations (Table 4 or 5 in the Additional file 1, respectively). The variance contributed by all values of a particular annotation was added up to the variance of the annotation in order to rank the annotations (Table 1 to 3 in the Additional file 1). Each of the annotation values is a centroid of all experimental conditions annotated by this value. These conditions may cluster densely around their centroid, well-separated from all other conditions, or they may show inhomogenous transcription, overlapping with conditions annotated by another value. This can be assessed by the SV, which is high for the former and low for the latter case. Computation of a SV for a certain cluster of elements is based on the comparison of its tightness, and its separation from neighboring clusters. We computed it on the basis of the χ^2 ^distances and used it as a second criterion for ranking and filtering.

### Significance analysis of differences between two traits

Significance analysis of microarrays (SAM) has been performed according to standard procedures (detailed in Additional file 1) to assess the difference between neighboring traits.

## Authors' contributions

KF, CB, SW, and JD contributed to the methodological development of the in-silico part of the study. KF provided the basic idea, implemented the majority of the methods, performed most of the analyses and wrote the manuscript. AB, BB, and NCH provided the microarray data analyzed in this study, JH contributed to concept and experimental design. AB, BB, NCH, MF and OW contributed with biological or medical expert knowledge for discretization and biological interpretation. OW and MF helped to draft the manuscript. All authors read and approved the final manuscript.

## Supplementary Material

Additional File 1 **Supplemental material**. Details in form of additional text, tables and figures. Click here for file
