# Supplementary material for: Systematic interpretation of microarray data using experiment annotations
Source: BMC Genomics. 2006 Dec 20;7:319. doi: 10.1186/1471-2164-7-319 (PMC1774576; doi:10.1186/1471-2164-7-319)
Supplement: Additional File 1 — Supplemental material. Details in form of additional text, tables and figures. [file 1471-2164-7-319-S1.pdf]

Supplement to:
Systematic interpretation of microarray data using experiment annotations

Kurt Fellenberg, Christian H. Busold, Olaf Witt, Andrea Bauer,
Boris Beckmann, Nicole C. Hauser, Marcus Frohme, Stefan Winter,
Jürgen Dippon, and Jörg D. Hoheisel

Detecting analysis-interfering artifacts and pitfalls in experimental design

Analysis-interfering artifacts (yeast data). The Eurofan II - Node B2 project [1, 2, 3, 4, 5, 6, 7] was an enterprise to study the *S. cerevisiae* transcriptome under various experimental conditions (such as high and low glucose concentrations, calcium shortage or heat shock) on a consistent platform. The investigated dataset comprises 229 out of 253 hybridizations performed by five European groups using whole-genome nylon membrane macroarrays and radioactive label. All experiments were performed using the same type of array, suggesting that they should all be comparable.

However, combining all the data in one analysis and ranking the experiment annotations by their variance, we obtain Table 1. It shows which of the numerous parameters correlate to transcription in this experimental context. Unfortunately, the total variance is dominated by the array production batch ('array series'). We generally observed this artifact to distort otherwise revealing radioactive data [8]. The variance introduced by the different array batches interferes with comparing the yeast hybridizations in a larger context. Because array batch is in the first place, we do not present any other aspect of these data in this paper.

Pitfalls in experimental design (fly data). A high-ranking technical parameter should be regarded a warning sign more than a death sentence to further anaysis of the datset, though. In the next example, the variance captured by array series may comprise a considerable share of biological variance due to a flaw in the experimental design. The data summerized by Table 2 draw a picture of the entire drosophila life cycle. They are described in detail by ref. [9].

Out of 120 experiment annotations, only 7 take different values throughout the dataset. These data stem from the more common glass-microarrays involving fluorescent labelling. Here, we would expect much less variance corresponding to different array batches because ratios reflecting the competition of two differently labelled transcripts should be less dependent on the absolute amount of binding sites, i.e. on the amount of DNA spotted. In contrast, the array batch shows up on rank 4. But performing these hybridizations, array batches have not been evenly distributed over the development of the fly. In contrast, array series 2 was only used for embryonic stages, for example. Biological variance acts as a confounder and might well make up a considerable

amount of the variance captured by array series.

A second flaw in the experimental design becomes visible in the rank of the annotation array individual. This variable offers the opportunity to track fabrication faults such as pins sticking in an upper position in the pin tool not touching the surface for a limited number of consecutively produced chips. The numbering of individual arrays follows the chronological order of their fabrication. But since they were largely used in the same sequence, it also reflects the chronological order of the hybridization experiments. Thus consecutive blocks of array individuals may as well correspond to experiment clusters representing particular experimental conditions rather than to a fabrication fault. Arrays 3 to 6 correspond to very similar pupal stages two and three, just to name one example (data not shown).

Thus, biological variance acts as a confounder for technical annotations, indicating flaws in experimental design rather than an artifact. Although it cannot be shown in this data example, we generally assume that the array batches have little influence on two-channel data. The remaining annotations have been investigated by CA. They draw a sound and revealing picture of the drosophila embryonic development (data not shown). In order to eliminate the confounding influence of the hybridization order, the arrays should be randomly permuted before use.

Overview (cancer data)

The pancreas cancer data comprise 87 hybridizations of 20 pancreas carcinoma and 10 normal tissue samples stemming from 30 patients. The chip comprises 3559 features (in double-spotting) with the main focus on cancer-relevant genes [10]. The annotation explaining most variance of the transcription data is the tumor type (Table 3). As for the previous data set, these data show a non-random distribution of array production batches among the experimental conditions, albeit to a lesser extent. Ranking the annotations by contributed variance (inertia), array series shows up at position 10. It shows a negative SV, though. The variance is due to an unbalanced distribution of the array batches throughout the tumor types. Ductal adenocarcinoma have been exclusively hybridized on array batch 8 just to give one example. Excluding such unique tumor types as well as unbalanced array batches shifts array series to a rank at the lower part of the table (Table 6), corroborating our assumption that direct comparisons across array batches are possible with two-channel data.

Thus, we further investigated the pancreas samples. They have been summarized by reducing to only four trait-clusters (Fig. 2). The clusters are numbered by increasing malignancy. Figure 3 arranges the cluster-centroids from right to left, showing associated genes.

Benign. Many genes associated to cluster 1 (red) and 2 (blue) are indicative of normal, differentiated and functional pancreatic tissue: Pancreatic lipase (PNLIP) is a typical enzyme secreted by normal pancreatic exocrine cells in order to digest nutritional triglycerides. Carboxypeptidase A1 (CPA1) is a pancreatic

exopeptidase. The sequence of PRSS2 is similar to trypsinogen IVa precursor mRNA. Trypsinogen is a typical pro-enzyme secreted by normal pancreatic exocrine cells in order to digest nutritional proteins. Pancreatic polypeptide (PPY) is a pancreas specific hormone which is involved in the regulation of exocrine pancreas secretion and biliary tract mobility [11]. The Myeloid cell leukemia sequence 1 (MCL1) was found to be expressed in normal fetal and embryonic pancreatic tissues and has been suggested to be involved in the control of proliferation and differentiation of normal pancreatic cells [12] and appears to play a role in control of pancreatic islet cell growth [13]. Taken together, these genes are typically expressed by normal pancreatic cells and encode for proteins required for food digestion.

Mucinous. Exclusively associated to cluster three (magenta, mucinous tumors) are the connective tissue growth factor (CTGF) which will be discussed in context of present alcohol consumption as well as the glutathione peroxidase 3 (GPX3) which was reported to be overexpressed in ovarian cancer [14]. CTGF is expressed as a 2.4-kb mRNA in a broad spectrum of human tissues. Sequence comparison revealed that CTGF belongs to a group known as the immediate-early genes, which are expressed after induction by growth factors or certain oncogenes [15]. Connective tissue growth factor is involved in pancreatic repair and tissue remodeling in acute necrotizing pancreatitis [16].

Mucinous and ductal. On the left side of the plot we find genes associated both to cluster 3 and 4: Pancreatic cancer shows a strong desmoplastic reaction characterized by a remarkable proliferation of interstitial connective tissue (collagen, fibronectin (FN1)) [17, 18, 19]. The tyrosine kinase receptor (Tie-1) was shown to be upregulated in (and can serve as a prognostic marker for) various metastatic malignancies incl. leucemia, breast and gastric cancer [20, 21, 22, 23, 24, 25, 26, 27]. However, to our knowledge, we are the first to report it in the context of pancreas carcinoma. The interferon-alpha induced 11.5 kDa protein (IFI27) was suggested to be a novel marker of epithelial proliferation and cancer [28] and will be discussed later on in the context of past alcohol consumption. The nonspecific cross-reacting antigen (NCA) is already associated more to cluster four (adenocarcinoma), confirming results reported earlier [29].

Ductal. Exclusively associated to the fourth cluster (green, highest malignancy) are the fibroblast growth factor 2 (FGF2, consistent with [30]), the *Clostridium perfringens* enterotoxin receptor (CPE-R) which was discussed in context of prostate cancer [31], and Glutathione [32]. Also applicable to discriminating the highly agressive tumors from the mucinous are mucin 1 (MUC1), which is in agreement with [33], as well as two more genes described as follows. Increasing evidence has accumulated in support of the hypothesis that growth hormone (GH) and insulin-like growth factors (IGFs) play a role in carcinogenesis. Insulin like growth factor binding protein 3 (IGFBP3) is upregulated in pancreatic endocrine tumors and its overexpression is significantly more com-

mon in metastatic disease [34]. High expression of IGFBP3 has been associated with invasiveness and poor prognosis in other cancer types [35]. GH receptor antagonist treatment decreased colon carcinoma growth in nude mice, associated with reduction in circulating IGFBP3 levels [36]. Elongation factor 1γ (EF1γ) is overexpressed in esophageal cancer with severe lymph node metastasis and far advanced stages of the disease compared with non-overexpressing cases [37]. In summary, genes affiliated to cluster four are known to be associated with metastasis, advanced stage disease and poor prognosis of pancreatic and other cancers.

Cluster four (cancer data)

To demonstrate how the analysis prodeeds towards more detail, we now analyze cluster four alone. It comprises six annotation values that are combined to four (Fig. 6) gaining perfect projection within two dimensions (Fig. 7, upper right). Figure 7 shows the variance within cluster four. All 22 measurements annotated by traits of cluster four (black boxes) are related to tumors classified as either ductal adenocarcinoma or other (i.e. none of the more benign tumor types). For 16 measurements, patients died within one year after surgery.

Pink cluster. The upper left corner holds six measurements annotated with no other than these traits along with genes associated with normal pancreatic function (pancreatic polypeptide PPY and Glycogen synthase) or adhaesion (laminin alpha 5, LAMA5) along with all other measurements not annotated with any trait of cluster four (grey boxes).

Blue cluster. All other 16 measurements annotated by cluster four are metastatic (tumor site=kidney). 12 of them stem from patients who received post operational chemotherapy. They split up into 8 measurements annotated with both past alcohol consumption and chemotherapy preceeding surgery (red) and 8 others classified pN stage 1 as well as WHO stage III (green). Common to both groups is an overexpression of mucin 1 (MUC1, [33]), GW112 (reported in context of gastric cancer metastasis [38]), and the S100 calcium binding protein (S100Ca+) which was shown to promote invasiveness of pancreatic cancer [39].

Red cluster. Chemokine (CXC motif) receptor 4 (CXCR4) has been linked to metastazation, male gender and older ages in colorectal cancer [40] and has been reported in context of invasiveness of pancreas cancer before. Our data show it associated to past alcohol consumption as will be detailed later under Alcohol Consumption.

Green cluster. Another kind of ductal adenocarcinoma involving lymphnode invasion (pN stage=1) is associated to upregulation of Paxillin (PXN) and Fas-activated serine/threonine kinase (FASTK), instead. While PXN has been linked to cancer cell migration [41], FASTK might correspond to a gain of chromosome 7 as reported in context of radiation resistance in glioblastoma

multiforme [42].

In summary, trait cluster four annotates six non-metastatic and sixteen metastatic measurements. The latter fall into two transcriptional types, presumably due to cytogenetic rearrangements. From this step (Figs. 6 and 7), the analysis would continue by assessing the difference between the two annotation values shown in pink in Fig. 6 in another step. A last step would be to account for the difference between the two blue traits, completing the analysis of the most malign quarter of the pancreas data's variance.

Alcohol consumption (cancer data)

Knowing from Table 3 which parameters correlate to transcription, one can select one or several of special interest. Figure 4 projects the values taken by experiment annotation 'alcohol consumption' (Table 4). It explains 92.8% of their variance. The abscissa (first principal axis) largely projects the difference between the pooled normal tissue samples as a reference and alcohol intake of cancer patients. Because the reference samples have all been annotated as pooled instead of discriminating between present, past and no alcohol consumption also for healthy individuals, above difference is trivial, including also the difference between normal and cancer tissue under a different name.

Thus, on the right side of Fig. 4 there are tagged genes already discussed for the overview such as PRSS2, PNLIP, and PPY, which are typically expressed by normal pancreatic cells for food digestion and which are downregulated upon alcohol consumption (both past and present).

Past and present alcohol consumption. The following genes are upregulated with both past and present alcohol consumption: Fibronectin (FN1), and collagens Type I (COL1A2) and III (COL3A1) have already been discussed above in context of the strong desmoplastic reaction of pancreatic cancer. Matrix Metalloproteinase 2 (MMP2) has been found to be expressed in pancreatic cancers and has been positively correlated with metastasis [43, 44]. Furthermore, MMP2 has been found to be a diagnositc marker for pancreatic carcinoma in pancreatic juice [45].

In summary, the geneset negatively or positively associated to alcohol consumption in general characterizes healthy pancreatic tissue on one hand and the dense connective tissue reaction of pancreatic cancer involving fibronectin and collagens type I and III on the other.

Significant difference. In the following, we attempt to discriminate between present and past alcohol consumption among the cancer patients. The ordinate (second principal axis) explains 19.8% of the total variance, almost exclusively corresponding to the variance between past and present alcohol consumption. But is it also significant? We performed a significance analysis of microarrays (SAM, [46]). A dataset including all the genes (3559) and one gene-wise median per patient yielded (Δ=0.72) 1082 significant genes with an estimated false

discovery rate (FDR) of 4.5%. In rare cases, the technical variance of the measurement process may lower the number of significant genes. Here, however, including all hybridizations (double-spots averaged) instead of one median per patient yields more significant genes (1495 with 3.8% FDR at Δ=0.8), as may be expected (for reasonably reproducible measurements).

Past alcohol consumption. The two categories have been further characterized in terms of prior knowledge about single differential genes. Following genes are exclusively associated to past alcohol consumption, all previously reported in context of carcinogenesis or chronic pancreas damage:

IFI27, also associated to cluster 3 and 4 in Fig. 3 is an interferon alpha-inducible protein. It has very recently been found to be upregulated in epidermal skin lesions, during wound reapair in proliferating cells as well as in cutaneous squamous cell cancers and thus, it was suggested that IFI27 is a novel marker of epithelial proliferation and cancer [28]. The gene was found to be overexpressed in colon samples from patients with inflammatory bowel disease compared with normal colon samples [47] and in hepatocellular carcinoma samples [48]. Thus, increased expression of IFI27 may indicate chronic inflammation, regeneration and tissue repair of the pancreas in individuals with past alcohol consumption. All these physiological processes predispose to cancer development.

S100P is the calcium binding protein of protein family 100. S100 proteins are localized in the cytoplasm and/or nucleus of a wide range of cells, and involved in the regulation of a number of cellular processes such as cell cycle progression and differentiation. S100P has been found to stimulate cell proliferation and survival in an autocrine manner in cells [49]. Furthermore, it is implicated in pancreatic tumorigenesis and metastasis [50]. Members of the family of S100 proteins have been found to be expressed in various metastatic adenocarcinomas [51] and poorly differentiated carcinomas [52]. Our data show that alcohol consumption over a longer period is associated with expression of S100P in pancreatic cells, which may promote malignant transformation by chronic stimulation of cell proliferation and survival, making cells more prone to additional mutagenic events.

Keratins represent a family of more than 20 different polypeptides which are important markers of epithelial cell differentiation. Precursor cells in different tissues display high Keratin 19 (K19) levels, and upon differentiation, K19 expression becomes epithelial cell-specific. Many premalignant and malignant tissues display K19 expression, such as dysplasia and carcinoma of squamous epithelia and adenocarcinoma of the lung, breast, pancreas, stomach and colon [53]. Exocrine acinar cells and endocrine islet cells are well-differentiated cells which express the keratin combination 8 and 18, whereas the less-differentiated cells of the ductal tree are characterized by the additional expression of keratin 7 and keratin 19 [54]. In the developing pancreas, duct-like precursor cells harbor high K19 expression [53]. Cytokeratin 19 expression has been described as expression marker in pancreatic carcinomas [55]. Higher expression of cytokeratin 19 was observed in human pancreatic epithelia in early stages of development (14 weeks of gestation) compared with adult tissues [56]. Thus, past alcohol

consumption is associated with expression of a marker gene for undifferentiated, immature stem-cell like pancreatic cells. This may reflect chronic damage of panreatic cells with subsequent regenerative activity of the organ which in turn can render pancreatic cells more prone to additional genetic mutational changes.

Also associated to past alcohol consumption is the chemokine receptor 4 (CXCR4). Tumour cell migration and metastasis share many similarities with leukocyte trafficking, which is critically regulated by chemokines and their receptors. The chemokine receptor 4 is highly expressed in primary and metastatic human breast cancer cells but is undetectable in normal mammary tissue and has been implicated in breast cancer metastasis [57]. Similarly, CXCR4 has been implicated in thyroid carcimoma invasion and tumor cell migration [58]. In pancreatic carcinoma, the CXCR4 receptor ligand system may play a role in the pancreatic cancer progression through tumor cell migration and invasion [59, 60]. Thus, past alcohol consumption is associated with expression of a receptor system that has been implicated in pancreatic cancer cell migration, invasion and metastasis.

In summary, genes upregulated with past alcohol consumption have been linked to pysiological processes associated with increased risk for malignant transformation and genes involved in pancreatic cancer cell proliferation, survival, invasion, metastasis, and impaired cell differentiation.

Present alcohol consumption. Following five genes associated to present alcohol consumption are tagged in Figure 4:

The connective tissue growth factor (CTGF) is expressed as a 2.4-kb mRNA in a broad spectrum of human tissues. Sequence comparison revealed that CTGF belongs to a group known as the immediate-early genes, which are expressed after induction by growth factors or certain oncogenes [15]. Connective tissue growth factor is involved in pancreatic repair and tissue remodeling in acute necrotizing pancreatitis [16].

The vascular endothelial growth factor (VEGF) is a mitogen primarily for vascular endothelial cells. It is upregulated in many cancer tissues and plays a central role in tumor angiogenesis [61]. VEGF-expression is induced in endothelial cells after ethanol exposure *in vitro* [62] and in gastric mucosa *in vivo* [63]. Increased VEGF-expression was found upon moderate ethanol consumption in rat skeletal muscle [64]. Thus, these reports support our finding that present alcohol consumption induces VEGF-expression in pancreatic tissue and may promote tumor-angiogenesis.

The tissue inhibitor of metalloproteinase 3 (TIMP3) is an inhibitor of matrix-metalloproteinases and an important regulator of inflammatory responses [65]. Increased metalloproteinase-activities are found in experimental and acute pancreatitis [66, 67]. Thus, increased expression of TIMP3 in individuals with present alcohol consumption may indicate the acute response of the pancreas to cope with ethanol induced tissue damage of the organ.

In rats, ethanol intake leads to increased secretion of tissue inhibitor of metalloproteinase 2 (TIMP2) by pancreatic stellate cells (PSCs) which are thought

to play a central role in pancreatic extracellular matrix formation and fibrogenesis [68]. Imbalance of expression of matrix metalloproteinases (MMPs) and their inhibitors including TIMP2 was described in human pancreatic carcinoma [69]. In our findings, the gene is less specific for present alcohol consumption. We mention it because of the direct experimental evidence for being linked to ethanol intake.

Also as overexpressed specifically with present alcohol consumption we observe the interferon induced transmembrane protein 1 (IFITM1). Interferon expression is increased in patients with acute pancreatitis compared with chronic pancreatitis [70], corroborating our assumption that we can discriminate between acute and past alcohol consumption in the context of pancreas cancer development.

Nine more genes of similar specificity, i.e. showing at least higher abundance with present than with past alcohol consumption, are encircled in grey in Figure 4:

Expression of ETS2 (v-ets erythroblastosis virus E26 oncogene homolog 2 (avian)), a protooncogene and transcription factor, occurs in a variety of cell types. Translocation in acute myeloid leukemia M2 involves the ETS2 gene [71].

Thrombospondin I (THBS1) is a multimodular secreted protein that associates with the extracellular matrix and possesses a variety of biological functions, including a potent angiogenic activity. Thrombospondin 1 has been implicated in tumor invasion, angiogenesis and metastasis of pancreatic carcinoma [72] and is a predictor of ductal pancreatic carcinoma [73].

Thymidylate synthetase (TYMS) is involved in maintaining the dTMP (thymidine-5-prime monophosphate) pool critical for DNA replication and repair. The enzyme has been of interest as a target for cancer chemotherapeutic agents. It is considered to be the primary site of action for 5-fluorouracil, 5-fluoro-2-prime-deoxyuridine, and some folate analogs. TYMS expression is a marker of poor prognosis in resected pancreatic cancer. Patients with high intratumoral TYMS expression benefit from adjuvant therapy with 5-fluorouracil [74].

For the protein NM23B expressed in non-metastatic cells 2, abundance in human pancreatic cancer is positively associated with lymph node metastasis, perineural invasion and poor prognosis [75].

The corticotropin-releasing hormone system regulates the mammalian stress response by coordinating the activity of the hypothalamic-pituitary-adrenal axis. The corticotropin releasing hormone binding protein (CRHBP) is an important element in the CRH system.

The early growth response 2 (EGR2) protein induces apoptosis in various cancer cell lines [76] and may be involved in the tumor growth suppressing effect of the PTEN pathway [77]. To our knowledge we are the first to report it as overexpressed with alcohol consumption or in pancreas carcinoma.

The polo-like kinase 1 (PLK1) encodes a protein serine/threonine kinase which plays a role in cell cycle regulation. Expression of PLK1 promotes mitosis (cell proliferation) and cells transformed with PLK1 grow in soft agar and produce tumors in nude mice. Therefore PLK may be involved in the promo-

tion or progression of cancers [78]. Elevated expression of PLK1 occurs in many different types of cancer, and PLK1 has been proposed as a diagnostic marker for several tumors. siRNA-mediated knock-down of PLK1 dramatically inhibited cell proliferation, decreased viability, and resulted in cell-cycle arrest and apoptosis [79]. Very recently, Plk1 mRNA was found to be overexpressed in pancreatic cancer cell lines and in human tumors. Depletion of Plk1 in pancreatic cancer cells by the use of antisense oligonucleotides induced cell cycle arrest in G2-M as well as a drastic reduction in proliferation rates. It was suggested that Plk1 is a potential therapeutic target for the treatment of pancreatic cancer [80].

The oncogene RHOA (ras homolog gene family, member A) was found frequently overexpressed in gastric cancer tissues and cells compared with normal tissues or gastric epithelial cells. Both RhoA-specific siRNA and dominant-negative RhoA expressions could significantly inhibit the proliferation and tumorigenicity of AGS cells [81]. RhoA overexpression has been linked to cancer cell detachment and metastasis [82] and to progression of testicular cancer [83].

The dual specificity phosphatase 1 (DUSP1) is higly induced by enviromental stress [84]. It has dual specificity for tyrosine and threonine and specifically inactivates mitogen-activated protein kinase in vitro [85].

In summary, present alcohol consumption is associated with expression of genes that control cell proliferation and transformation of pancreatic cells, DNA-synthesis, encode for oncogenes, genes linked to acute inflammatory and stress responses, tumor angiogenesis, pancreas carcinoma metastasis, pancreatic repair and tissue remodeling, and pancreatic extracellular matrix composition.

In contrast to past alcohol consumption, present alcohol intake is associated with expression of immediate response genes to tissue damage, repair and remodeling, inflammatory and stress response (IFITM1, CRHBP, TIMP 2 and 3, DUSP1, CTGF). Additionally, correspondence analysis also identified genes that are involved in control of cell proliferation, oncogenesis and tumor angiogenesis to be upregulated in present alcohol intake.

Preprocessing of transcription data

Sampling, labeling, hybridization, scanning and imaging were performed as described [2, 3, 4, 5, 6, 7, 9, 10].

The raw intensity values have been normalized based on a robust affine-linear regression of one measurement versus a control measurement. The method is described in refs. [86] and [87] and performs better than or equally to lowess normalization [88]. For the monochannel (yeast) data, each measurement was normalized versus the genewise median of the 75 hybridizations of the control condition, resulting in absolute intensities. For the two-channel data (fly, human cancer), the channel belonging to the control condition served to normalize the other channel of the same hybridization, resulting in intensity ratios [8].

Subsequently, genes have been filtered for considerable absolute intensity level in at least one of the conditions (i); reproducibility in the separation from

the control condition in at least one of the other conditions (ii) [86, 87, 8]; and lack of saturation (iii). To compute intensity levels (i) from multichannel ratios, these ratios have had been multiplied with the genewise median of the absolute values of the control channels [8]. Following thresholds have been applied:

For the yeast data, the condition-median had to be at least 1.5×10^4 in at least one condition (i). Furthermore, we asked for complete separation (min-max separation, [86]) of control and non-control measurements in at least one condition (ii). 924 out of 6103 genes satisfied both criteria.

For the fly data, we filtered genes showing condition-median intensity equal or greater 10^5 in at least one condition (i), as well as an average minmax separation greater or equal to zero, yielding 6938 out of 22429 genes (ii).

For the human pancreatic cancer data, condition-median intensity had to be at least 2×10^5 in at least one condition (i) accompanied by positive minmax separation in at least one condition (ii). Furthermore, because the data were prone to saturated spots, we excluded all genes with **raw** intensities exceeding 3×10^6 in any measurement (iii), leaving 442 out of 3559 genes.

Further analysis was based on intensities or log ratios of the surviving genes for mono- (yeast) or multi-channel (fly and cancer) data, respectively. Experiment annotation values were represented by adding the condition medians of the annotated conditions.

Preprocessing of experiment annotations

Much like the transcription data themselves, their annotations need to be preprocessed. Not all annotated traits correlate with transcription. Moreover, even before being subject to judgement in this respect, some traits need to be at all defined. The values taken by a continous annotation will most probably differ for all measurements (if only in the third position after the decimal point). In order to characterize groups of measurements, the value range has to be discretized into few values (intervals) that can be discriminated on the basis of the collected data.

Discretization.

All annotations taking more than 4 values were subjected to discretization. The individual decisions for the presented data are detailed below.

For the yeast data, annotations ‘array individual’, ‘total activity’, ‘date of entry month’, ‘experimentator hybridization’, and ‘temperature’ were discretized as shown in Fig. 8. All values were kept without grouping for ‘array series’, ‘strains’, ‘transgenes’, ‘base media’, ‘temporary additive’, ‘temporary additive conc.’, and ‘glucose’. Annotations ‘label incorporation rate’, ‘exposure time’, and ‘date of entry day’ where inactivated for obviously not showing meaningful value groups. ‘incubation period’ may better be investigated additive by additive and was therefore inactivated in this context. Likewise, ‘temperature shift incubation period’ may better be investigated temperature by temperature and was therefore inactivated. ‘array hybridization’ exhibited clusters, but we suspect that some experimenters did not annotate it according to its correct

meaning (otherwise, it would mean that one chip was reused up to 14 times). As a precaution, it was inactivated, as well.

For the fly data, annotation 'array individual' was discretized as shown in Fig. 9, 'embryo' was kept unchanged, and 'label incorporation rate' as well as 'amount of cDNA' were inactivated for showing no obvious clustering of consecutive values.

For the cancer data, annotations 'live status', 'tumor type', 'pT stage', 'tumor subregion', 'smoking', 'alcohol consumption', 'weight loss in last 4 weeks', and 'OP procedure' were discretized as shown in Fig. 10. Initially, we kept all values of 'tumor type' for appearing nicely correlated to transcription. However, IPMT samples showed negative silhouette values because they do not separate from the healthy tissues (data not shown). Annotations 'array series', 'tumor size', and 'WHO stage' were taken on unchanged. Annotations 'array individual, 'birth date day', 'birth date month', 'birth date year', and 'CA 19-9' were inactivated for showing no obvious clustering of consecutive values.

Filtering. After discretization, annotation values are projected as centroids of the according experimental conditions. This type of investigation works the better, the more the projected traits vary in their transcription profiles. Also, each annotation value should correspond to a homogenous cluster of conditions well-separated from conditions annotated with other values of the same annotation. In this case, it appears perfectly justified to briefly characterize it by a single data point, which can be regarded a prototype or class-representative for the particular annotation value.

But not all traits should be taken at face value. Some do not carry considerable amounts of information in terms of transcription behaviour. We assess this by computing their inertia contributions. The inertia, computed as the χ^2 statistic devided by the grand total of the data table, is a means of assessing the variance or information content of a data table. Here, each table column contains the (prototype) transcription profile of a particular experiment annotation value, contributing a certain share to the total inertia of the table. The discretized annotation values are ranked and/or filtered according to the variance they contribute either in the context of the values of only one (Tab. 4) or all annotations (Tab. 5). For the latter, the variance contributed by all values of a particular annotation can be added in order to rank the annotations by their "relevance" (Tabs. 1, 2, 3, and 6).

To assess if a trait annotates a distinct cluster of experiments or not, we compute the Silhouette value (SV, [89]). Let there be an experimental annotation $\mathbf{A}$ taking values $i \epsilon \mathbf{A}$. One SV per annotation value i and measurement j is computed as $s_{ij} = (b_{ij} - a_{ij})/max(a_{ij}, b_{ij})$, where a_{ij} is the average distance of annotated measurement j to all other measurements annotated with i and b_{ij} is the minimum of average distances of measurement j to all measurements not annotated with i. Here, the Silhouette scores were computed on the basis of the χ^2 distances.

A SV close to one will result for measurements well-separated from the measurements of neighboring clusters (composed of measurements annotated with

annotation values other than i). A score around zero means that the measurement could be assigned to another annotation value, as well. A score close to minus one denotes that the object is most likely misclassified, i.e. transcriptionally affiliated to another but the annotated annotation value.

The average SV of all measurements annotated with a particular annotation value i is used to rank and/or filter the annotation values (Tabs. 4 and 5). We further calculate the mean of the SV of all $i \epsilon \mathbf{A}$. The average SV for an annotation $\mathbf{A}$ (Tabs. 3 and 6) indicates if this parameter correlates with transcriptional changes in a reproducible manner.

Figure 4 exemplifies the typical behaviour of an uninformative annotation value. The value 'never' shows a negative SV because it is dispersed over the area spanned by the remaining three values of 'alcohol consumption'. It also shows a low variance contribution, mainly because it is quite evenly spread out.

In order not to let the principal axes be attracted either by such ubiquitously dispersed features or those showing an "average" transcription profile, these should be thoroughly filtered. Figure 4 shows informative and uninformative values combined in a single annotation, showing that it is advisable to filter out single annotation values rather than whole annotations. When visualizing more than one parameter (Figs. 2 and 3), we disregarded all annotation values showing negative SV or inertia contributions below one percent.

Whereas the former warrants tight clustering of measurements annotated by a particular annotation value, the latter, in addition to picking marked transcription profiles, also selects for a substantial amount of observations per annotation value. Out of the annotation values listed in Tab. 5 that show positive SV, all consisting of less than four measurements have been excluded from visualization by their low inertia contributions. This is because adding fewer measurements causes a lower column weight and will therefore result in a smaller inertia-contribution. Whereas the inertia criterion favours the annotations having many values, the SV does the opposite. Both criteria supplement each other in order to identify traits potent to characterize the transcription data under study.

Significance analysis of differences between two traits

Significance analysis of microarrays (SAM) has been performed to assess the difference between past and present alcohol consumption. It has been based on log_2 transformed ratios, the data table containing all the genes on the array. Initially, the table contained all measurements of all patients affiliated to present and past alcohol consumption, as well. In order to exclude the technical variance, the gene-wise median of all measurements for one patient has been computed, subjecting one column per patient to SAM as two-class (past and present), unpaired data. A second analysis was performed including the technical variance. Here, we only averaged over the two measurements stemming from duplicate spots on the same array beforehand. SAM version 1.21 (Nov 2002, Excel-plugin obtained from [90]) was executed with seed 67420160 and

1000 permutations for both cases.

References

[1] **Eurofan II - Node B2** http://mips.gsf.de/proj/eurofan/eurofan_2/b2.

[2] Becerra M, Lombardia-Ferreira L, Hauser N, Hoheisel J, Tizon B, Cerdán M: **The yeast transcriptome in aerobic and hypoxic conditions: effects of hap1, rox1, rox3 and srb10 deletions.** *Mol Microbiol* 2002, **43**(3):545–55, http://eutils.ncbi.nlm.nih.gov/entrez/eutils/elink.fcgi?cmd=prlinks\&db\%from=pubmed\&retmode=ref\&id=11929514.

[3] Hayes A, Zhang N, Wu J, Butler P, Hauser N, Hoheisel J, Lim F, Sharrocks A, Oliver S: **Hybridization array technology coupled with chemostat culture: Tools to interrogate gene expression in Saccharomyces cerevisiae.** *Methods* 2002, **26**(3):281–90, http://eutils.ncbi.nlm.nih.gov/entrez/eutils/elink.fcgi?cmd=prlinks\&db\%from=pubmed\&retmode=ref\&id=12054884.

[4] Lombardia L, Becerra M, Rodriguez-Belmonte E, Hauser N, Cerdán M: **Genome-wide analysis of yeast transcription upon calcium shortage.** *Cell Calcium* 2002, **32**(2):83–91, http://eutils.ncbi.nlm.nih.gov/entrez/eutils/elink.fcgi?cmd=prlinks\&db\%from=pubmed\&retmode=ref\&id=12161108.

[5] Lagorce A, Hauser N, Labourdette D, Rodriguez C, Martin-Yken H, Arroyo J, Hoheisel J, Francois J: **Genome-wide analysis of the response to cell wall mutations in the yeast Saccharomyces cerevisiae.** *J Biol Chem* 2003, **278**(22):20345–57, http://eutils.ncbi.nlm.nih.gov/entrez/eutils/elink.fcgi?cmd=prlinks\&db\%from=pubmed\&retmode=ref\&id=12644457.

[6] Yin Z, Wilson S, Hauser N, Tournu H, Hoheisel J, Brown A: **Glucose triggers different global responses in yeast, depending on the strength of the signal, and transiently stabilizes ribosomal protein mRNAs.** *Mol Microbiol* 2003, **48**(3):713–24, http://eutils.ncbi.nlm.nih.gov/entrez/eutils/elink.fcgi?cmd=prlinks\&db\%from=pubmed\&retmode=ref\&id=12694616.

[7] Becerra M, Lombardia LJ, Gonzalez-Siso MI, Rodriguez-Belmonte E, Hauser NC, Cerdán ME: **Genome-wide analysis of the yeast transcriptome upon heat and cold shock.** *Comp Funct Genom* 2003, **4**(4):366–375.

[8] Fellenberg K, Hauser N, Brors B, Hoheisel J, Vingron M: **Microarray data warehouse allowing for inclusion of experiment annotations in statistical analysis.** *Bioinformatics* 2002, **18**(3):423–33, http://eutils.ncbi.nlm.nih.gov/entrez/eutils/elink.fcgi?cmd=prlinks\&db\%from=pubmed\&retmode=ref\&id=11934741.

[9] Hild M, Beckmann B, Haas S, Koch B, Solovyev V, Busold C, Fellenberg K, Boutros M, Vingron M, Sauer F, Hoheisel J, Paro R: **An integrated gene annotation and transcriptional profiling approach towards the full gene content of the Drosophila genome.** *Genome Biol* 2003, **5**:R3, http://eutils.ncbi.nlm.nih.gov/entrez/eutils/elink.fcgi?cmd=prlinks\&db\%from=pubmed\&retmode=ref\&id=14709175.

[10] Esposito I, Bauer A, Hoheisel J, Kleeff J, Friess H, Bergmann F, Rieker R, Otto H, Kloppel G, Penzel R: **Microcystic tubulopapillary carcinoma of the pancreas: a new tumor entity?** *Virchows Arch* 2004, **444**(5):447–53, http://eutils.ncbi.nlm.nih.gov/entrez/eutils/elink.fcgi?cmd=prlinks\&db\%from=pubmed\&retmode=ref\&id=15014986.

[11] Schwartz T, Tager H: **Isolation and biogenesis of a new peptide from pancreatic islets.** *Nature* 1981, **294**(5841):589–91, http://eutils.ncbi.nlm.nih.gov/entrez/eutils/elink.fcgi?cmd=prlinks\&db\%from=pubmed\&retmode=ref\&id=7031480.

[12] Kobayash H, Doi R, Hosotani R, Miyamoto Y, Koshiba T, Fujimoto K, Ida J, Tsuji S, Nakajima S, Kawaguchi M, Shiota K, Imamura M: **Immunohistochemical analysis of apoptosis-related proteins in human embryonic and fetal pancreatic tissues.** *Int J Pancreatol* 2000, **27**(2):113–22.

[13] Matsushita K, Okita H, Suzuki A, Shimoda K, Fukuma M, Yamada T, Urano F, Honda T, Sano M, Iwanaga S, Ogawa S, ichi Hata J, Umezawa A: **Islet cell hyperplasia in transgenic mice overexpressing EAT/mcl-1, a bcl-2 related gene.** *Mol Cell Endocrinol* 2003, **203**(1-2):105–16.

[14] Hough C, Cho K, Zonderman A, Schwartz D, Morin P: **Coordinately up-regulated genes in ovarian cancer.** *Cancer Res* 2001, **61**(10):3869–76, http://eutils.ncbi.nlm.nih.gov/entrez/eutils/elink.fcgi?cmd=prlinks\&db\%from=pubmed\&retmode=ref\&id=11358798.

[15] Kim H, Nagalla S, Oh Y, Wilson E, Roberts C Jr, Rosenfeld R: **Identification of a family of low-affinity insulin-like growth factor binding proteins (IGFBPs): characterization of connective tissue growth factor as a member of the IGFBP superfamily.** *Proc Natl Acad Sci U S A* 1997, **94**(24):12981–6, http://eutils.ncbi.nlm.nih.gov/entrez/eutils/elink.fcgi?cmd=prlinks\&db\%from=pubmed\&retmode=ref\&id=9371786.

[16] di Mola F, Friess H, Riesle E, Koliopanos A, Buchler P, Zhu Z, Brigstock D, Korc M, Buchler M: **Connective tissue growth factor is involved in pancreatic repair and tissue remodeling in human and rat acute necrotizing pancreatitis.** *Ann Surg* 2002, **235**:60–7, http://eutils.ncbi.nlm.nih.gov/entrez/eutils/elink.fcgi?cmd=prlinks\&db\%from=pubmed\&retmode=ref\&id=11753043.

[17] Gress T, Muller-Pillasch F, Lerch M, Friess H, Buchler M, Adler G: **Expression and in-situ localization of genes coding for extracellular matrix proteins and extracellular matrix degrading proteases in pancreatic cancer.** *Int J Cancer* 1995, **62**(4):407–13, http://eutils.ncbi.nlm.nih.gov/entrez/eutils/elink.fcgi?cmd=prlinks\&db\%from=pubmed\&retmode=ref\&id=7635566.

[18] Lohr M, Trautmann B, Gottler M, Peters S, Zauner I, Maillet B, Kloppel G: **Human ductal adenocarcinomas of the pancreas express extracellular matrix proteins.** *Br J Cancer* 1994, **69**:144–51, http://eutils.ncbi.nlm.nih.gov/entrez/eutils/elink.fcgi?cmd=prlinks\&db\%from=pubmed\&retmode=ref\&id=8286197.

[19] Mollenhauer J, Roether I, Kern H: **Distribution of extracellular matrix proteins in pancreatic ductal adenocarcinoma and its influence on tumor cell proliferation in vitro.** *Pancreas* 1987, **2**:14–24, http://eutils.ncbi.nlm.nih.gov/entrez/eutils/elink.fcgi?cmd=prlinks\&db\%from=pubmed\&retmode=ref\&id=3554225.

[20] Lin W, Li A, Chi C, Chung W, Huang C, Lui W, Kung H, Wu C: **tie-1 protein tyrosine kinase: a novel independent prognostic marker for gastric cancer.** *Clin Cancer Res* 1999, **5**(7):1745–51, http://eutils.ncbi.nlm.nih.gov/entrez/eutils/elink.fcgi?cmd=prlinks\&db\%from=pubmed\&retmode=ref\&id=10430078.

[21] Verstovsek S, Estey E, Manshouri T, Keating M, Kantarjian H, Giles F, Albitar M: **High expression of the receptor tyrosine kinase Tie-1 in acute myeloid leukemia and myelodysplastic syndrome.** *Leuk Lymphoma* 2001, **42**(3):511–6, http://eutils.ncbi.nlm.nih.gov/entrez/eutils/elink.fcgi?cmd=prlinks\&db\%from=pubmed\&retmode=ref\&id=11699417.

[22] Tseng L, Hsu C, Wang H, Liu J, Chang H, Lo S, Wu C, Lui W, Chi C: **Tie-1 tyrosine kinase is an independent prognostic indicator for invasive breast cancer.** *Anticancer Res* 2001, **21**(3C):2163–70, http://eutils.ncbi.nlm.nih.gov/entrez/eutils/elink.fcgi?cmd=prlinks\&db\%from=pubmed\&retmode=ref\&id=11501841.

[23] Verstovsek S, Kantarjian H, Manshouri T, O'Brien S, Faderl S, Talpaz M, Cortes J, Albitar M: **Prognostic significance of Tie-1 protein expression in patients with early chronic**

phase chronic myeloid leukemia. *Cancer* 2002, **94**(5):1517–21, http://eutils.ncbi.nlm.nih.gov/entrez/eutils/elink.fcgi?cmd=prlinks\&db\%from=pubmed\&retmode=ref\&id=11920509.

[24] Yang X, Hand R, Livasy C, Cance W, Craven R: **Overexpression of the receptor tyrosine kinase Tie-1 intracellular domain in breast cancer.** *Tumour Biol* 2003, **24**(2):61–9, http://eutils.ncbi.nlm.nih.gov/entrez/eutils/elink.fcgi?cmd=prlinks\&db\%from=pubmed\&retmode=ref\&id=12853700.

[25] Karnani P, Kairemo K: **The new Tie-1 monoclonal antibodies detect angiogenesis in metastatic malignancies.** *Clin Cancer Res* 2003, **9**(10 Pt 2):3827S–30S, http://eutils.ncbi.nlm.nih.gov/entrez/eutils/elink.fcgi?cmd=prlinks\&db\%from=pubmed\&retmode=ref\&id=14506179.

[26] Ito Y, Yoshida H, Uruno T, Nakano K, Takamura Y, Miya A, Kobayashi K, Yokozawa T, Matsuzuka F, Kuma K, Miyauchi A: **Tie-1 tyrosine kinase expression in human thyroid neoplasms.** *Histopathology* 2004, **44**(4):318–22, http://eutils.ncbi.nlm.nih.gov/entrez/eutils/elink.fcgi?cmd=prlinks\&db\%from=pubmed\&retmode=ref\&id=15049896.

[27] Nakayama T, Hatachi G, Wen C, Yoshizaki A, Yamazumi K, Niino D, Sekine I: **Expression and significance of Tie-1 and Tie-2 receptors, and angiopoietins-1, 2 and 4 in colorectal adenocarcinoma: Immunohistochemical analysis and correlation with clinicopathological factors.** *World J Gastroenterol* 2005, **11**(7):964–9, http://eutils.ncbi.nlm.nih.gov/entrez/eutils/elink.fcgi?cmd=prlinks\&db\%from=pubmed\&retmode=ref\&id=15742397.

[28] Suomela S, Cao L, Bowcock A, Saarialho-Kere U: **Interferon alpha-inducible protein 27 (IFI27) is upregulated in psoriatic skin and certain epithelial cancers.** *J Invest Dermatol* 2004, **122**(3):717–21, http://eutils.ncbi.nlm.nih.gov/entrez/eutils/elink.fcgi?cmd=prlinks\&db\%from=pubmed\&retmode=ref\&id=15086558.

[29] Albers G, Fleuren G, Escribano M, Nap M: **Immunohistochemistry of CEA in the human pancreas during development, in the adult, chronic pancreatitis, and pancreatic adenocarcinoma.** *Am J Clin Pathol* 1988, **90**:17–22, http://eutils.ncbi.nlm.nih.gov/entrez/eutils/elink.fcgi?cmd=prlinks\&db\%from=pubmed\&retmode=ref\&id=3389342.

[30] Yamazaki K, Nagao T, Yamaguchi T, Saisho H, Kondo Y: **Expression of basic fibroblast growth factor (FGF-2)-associated with tumour proliferation in human pancreatic carcinoma.** *Virchows Arch* 1997, **431**(2):95–101, http://eutils.ncbi.nlm.nih.gov/entrez/

eutils/elink.fcgi?cmd=prlinks\&db\%from=pubmed\&retmode=ref\&id=9293890.

[31] Long H, Crean C, Lee W, Cummings O, Gabig T: **Expression of Clostridium perfringens enterotoxin receptors claudin-3 and claudin-4 in prostate cancer epithelium.** *Cancer Res* 2001, **61**(21):7878–81, http://eutils.ncbi.nlm.nih.gov/entrez/eutils/elink.fcgi?cmd=prlinks\&db\%from=pubmed\&retmode=ref\&id=11691807.

[32] Kennedy R, Konok G, Bounous G, Baruchel S, Lee T: **The use of a whey protein concentrate in the treatment of patients with metastatic carcinoma: a phase I-II clinical study.** *Anticancer Res* 1995, **15**(6B):2643–9, http://eutils.ncbi.nlm.nih.gov/entrez/eutils/elink.fcgi?cmd=prlinks\&db\%from=pubmed\&retmode=ref\&id=8669840.

[33] Adsay N, Merati K, Andea A, Sarkar F, Hruban R, Wilentz R, Goggins M, Iocobuzio-Donahue C, Longnecker D, Klimstra D: **The dichotomy in the preinvasive neoplasia to invasive carcinoma sequence in the pancreas: differential expression of MUC1 and MUC2 supports the existence of two separate pathways of carcinogenesis.** *Mod Pathol* 2002, **15**(10):1087–95, http://eutils.ncbi.nlm.nih.gov/entrez/eutils/elink.fcgi?cmd=prlinks\&db\%from=pubmed\&retmode=ref\&id=12379756.

[34] Maitra A, Hansel DE, Argani P, Ashfaq R, Rahman A, Naji A, Deng S, Geradts J, Hawthorne L, House MG, Yeo CJ: **Global expression analysis of well-differentiated pancreatic endocrine neoplasms using oligonucleotide microarrays.** *Clin Cancer Res* 2003, **9**(16 Pt 1):5988–95.

[35] Vestey SB, Perks CM, Sen C, Calder CJ, Holly JMP, Winters ZE: **Immunohistochemical expression of insulin-like growth factor binding protein-3 in invasive breast cancers and ductal carcinoma in situ: implications for clinicopathology and patient outcome.** *Breast Cancer Res* 2005, **7**:R119–29, http://dx.doi.org/10.1186/bcr963.

[36] Dagnaes-Hansen F, Duan H, Rasmussen LM, Friend KE, Flyvbjerg A: **Growth hormone receptor antagonist administration inhibits growth of human colorectal carcinoma in nude mice.** *Anticancer Res* 2004, **24**(6):3735–42.

[37] Mimori K, Mori M, Inoue H, Ueo H, Mafune K, Akiyoshi T, Sugimachi K: **Elongation factor 1 gamma mRNA expression in oesophageal carcinoma.** *Gut* 1996, **38**:66–70.

[38] Oue N, Aung P, Mitani Y, Kuniyasu H, Nakayama H, Yasui W: **Genes involved in invasion and metastasis of gastric cancer identified by**

array-based hybridization and serial analysis of gene expression. *Oncology* 2005, **69 Suppl 1**:17–22.

[39] Arumugam T, Simeone D, Golen KV, Logsdon C: **S100P promotes pancreatic cancer growth, survival, and invasion.** *Clin Cancer Res* 2005, **11**:5356–5364.

[40] Schimanski C, Schwald S, Simiantonaki N, Jayasinghe C, Gonner U, Wilsberg V, Jungingera T, Berger M, Galle P, Moehler M: **Effect of Chemokine Receptors CXCR4 and CCR7 on the Metastatic Behavior of Human Colorectal Cancer.** *Clinical Cancer Research* 2005, **11**:1743–1750.

[41] Crowe D, Ohannessian A: **Recruitment of focal adhesion kinase and paxillin to** ***beta*****1 integrin promotes cancer cell migration via mitogen activated protein kinase activation.** *BMC Cancer* 2004, **4**:18.

[42] Misra A, Pellarin M, Hu L, Kunwar S, Perhouse M, Lamborn K, Deen D, Feuerstein B: **Chromosome transfer experiments link regions on chromosome 7 to radiation resistance in human glioblastoma multiforme**. *Genes, Chromosomes and Cancer* 2006, **45**:20–30.

[43] Matsuyama Y, Takao S, Aikou T: **Comparison of matrix metalloproteinase expression between primary tumors with or without liver metastasis in pancreatic and colorectal carcinomas.** *J Surg Oncol* 2002, **80**(2):105–10, `http://eutils.ncbi.nlm.nih.gov/entrez/eutils/elink.fcgi?cmd=prlinks\&db\%from=pubmed\&retmode=ref\&id=12173379`.

[44] Hosotani R, Kawaguchi M, Masui T, Koshiba T, Ida J, Fujimoto K, Wada M, Doi R, Imamura M: **Expression of integrin alphaV-beta3 in pancreatic carcinoma: relation to MMP-2 activation and lymph node metastasis.** *Pancreas* 2002, **25**(2):e30–5, `http://eutils.ncbi.nlm.nih.gov/entrez/eutils/elink.fcgi?cmd=prlinks\&db\%from=pubmed\&retmode=ref\&id=12142752`.

[45] Yokoyama M, Ochi K, Ichimura M, Mizushima T, Shinji T, Koide N, Tsurumi T, Hasuoka H, Harada M: **Matrix metalloproteinase-2 in pancreatic juice for diagnosis of pancreatic cancer.** *Pancreas* 2002, **24**(4):344–7, `http://eutils.ncbi.nlm.nih.gov/entrez/eutils/elink.fcgi?cmd=prlinks\&db\%from=pubmed\&retmode=ref\&id=11961486`.

[46] Tusher V, Tibshirani R, Chu G: **Significance analysis of microarrays applied to the ionizing radiation response.** *Proc Natl Acad Sci U S A* 2001, **98**(9):5116–21, `http://eutils.ncbi.nlm.nih.gov/entrez/eutils/elink.fcgi?cmd=prlinks\&db\%from=pubmed\&retmode=ref\&id=11309499`.

[47] Dooley T, Curto E, Reddy S, Davis R, Lambert G, Wilborn T, Elson C: **Regulation of gene expression in inflammatory bowel disease and correlation with IBD drugs: screening by DNA microarrays.** *Inflamm Bowel Dis* 2004, **10**:1–14, http://eutils.ncbi.nlm.nih.gov/entrez/eutils/elink.fcgi?cmd=prlinks\&db\%from=pubmed\&retmode=ref\&id=15058520.

[48] Iizuka N, Oka M, Yamada-Okabe H, Hamada K, Nakayama H, Mori N, Tamesa T, Okada T, Takemoto N, Matoba K, Takashima M, Sakamoto K, Tangoku A, Miyamoto T, Uchimura S, Hamamoto Y: **Molecular signature in three types of hepatocellular carcinoma with different viral origin by oligonucleotide microarray.** *Int J Oncol* 2004, **24**(3):565–74, http://eutils.ncbi.nlm.nih.gov/entrez/eutils/elink.fcgi?cmd=prlinks\&db\%from=pubmed\&retmode=ref\&id=14767541.

[49] Arumugam T, Simeone D, Schmidt A, Logsdon C: **S100P stimulates cell proliferation and survival via receptor for activated glycation end products (RAGE).** *J Biol Chem* 2004, **279**(7):5059–65, http://eutils.ncbi.nlm.nih.gov/entrez/eutils/elink.fcgi?cmd=prlinks\&db\%from=pubmed\&retmode=ref\&id=14617629.

[50] Missiaglia E, Blaveri E, Terris B, Wang Y, Costello E, Neoptolemos J, Crnogorac-Jurcevic T, Lemoine N: **Analysis of gene expression in cancer cell lines identifies candidate markers for pancreatic tumorigenesis and metastasis.** *Int J Cancer* 2004, **112**:100–12, http://eutils.ncbi.nlm.nih.gov/entrez/eutils/elink.fcgi?cmd=prlinks\&db\%from=pubmed\&retmode=ref\&id=15305381.

[51] Herrera G, Turbat-Herrera E, Lott R: **S-100 protein expression by primary and metastatic adenocarcinomas.** *Am J Clin Pathol* 1988, **89**(2):168–76, http://eutils.ncbi.nlm.nih.gov/entrez/eutils/elink.fcgi?cmd=prlinks\&db\%from=pubmed\&retmode=ref\&id=2449069.

[52] Drier J, Swanson P, Cherwitz D, Wick M: **S100 protein immunoreactivity in poorly differentiated carcinomas. Immunohistochemical comparison with malignant melanoma.** *Arch Pathol Lab Med* 1987, **111**(5):447–52, http://eutils.ncbi.nlm.nih.gov/entrez/eutils/elink.fcgi?cmd=prlinks\&db\%from=pubmed\&retmode=ref\&id=2436593.

[53] Brembeck F, Rustgi A: **The tissue-dependent keratin 19 gene transcription is regulated by GKLF/KLF4 and Sp1.** *J Biol Chem* 2000, **275**(36):28230–9, http://eutils.ncbi.nlm.nih.gov/entrez/eutils/elink.fcgi?cmd=prlinks\&db\%from=pubmed\&retmode=ref\&id=10859317.

[54] Bouwens L: **Cytokeratins and cell differentiation in the pancreas.** *J Pathol* 1998, **184**(3):234–9, http://eutils.ncbi.nlm.nih.gov/entrez/

eutils/elink.fcgi?cmd=prlinks\&db\%from=pubmed\&retmode=ref\&id=9614373.

[55] Schussler M, Skoudy A, Ramaekers F, Real F: **Intermediate filaments as differentiation markers of normal pancreas and pancreas cancer.** *Am J Pathol* 1992, **140**(3):559–68, http://eutils.ncbi.nlm.nih.gov/entrez/eutils/elink.fcgi?cmd=prlinks\&db\%from=pubmed\&retmode=ref\&id=1372155.

[56] Kasper M, von Dorsche H, Stosiek P: **Changes in the distribution of intermediate filament proteins and collagen IV in fetal and adult human pancreas. I. Localization of cytokeratin polypeptides.** *Histochemistry* 1991, **96**(3):271–7, http://eutils.ncbi.nlm.nih.gov/entrez/eutils/elink.fcgi?cmd=prlinks\&db\%from=pubmed\&retmode=ref\&id=1917582.

[57] Muller A, Homey B, Soto H, Ge N, Catron D, Buchanan M, McClanahan T, Murphy E, Yuan W, Wagner S, Barrera J, Mohar A, Verastegui E, Zlotnik A: **Involvement of chemokine receptors in breast cancer metastasis.** *Nature* 2001, **410**(6824):50–6, http://eutils.ncbi.nlm.nih.gov/entrez/eutils/elink.fcgi?cmd=prlinks\&db\%from=pubmed\&retmode=ref\&id=11242036.

[58] Hwang J, Hwang J, Chung H, Kim D, Hwang E, Suh J, Kim H, You K, Kwon O, Ro H, Jo D, Shong M: **CXC chemokine receptor 4 expression and function in human anaplastic thyroid cancer cells.** *J Clin Endocrinol Metab* 2003, **88**:408–16, http://eutils.ncbi.nlm.nih.gov/entrez/eutils/elink.fcgi?cmd=prlinks\&db\%from=pubmed\&retmode=ref\&id=12519884.

[59] Koshiba T, Hosotani R, Miyamoto Y, Ida J, Tsuji S, Nakajima S, Kawaguchi M, Kobayashi H, Doi R, Hori T, Fujii N, Imamura M: **Expression of stromal cell-derived factor 1 and CXCR4 ligand receptor system in pancreatic cancer: a possible role for tumor progression.** *Clin Cancer Res* 2000, **6**(9):3530–5, http://eutils.ncbi.nlm.nih.gov/entrez/eutils/elink.fcgi?cmd=prlinks\&db\%from=pubmed\&retmode=ref\&id=10999740.

[60] Sato N, Fukushima N, Maitra A, Iacobuzio-Donahue C, van Heek N, Cameron J, Yeo C, Hruban R, Goggins M: **Gene expression profiling identifies genes associated with invasive intraductal papillary mucinous neoplasms of the pancreas.** *Am J Pathol* 2004, **164**(3):903–14, http://eutils.ncbi.nlm.nih.gov/entrez/eutils/elink.fcgi?cmd=prlinks\&db\%from=pubmed\&retmode=ref\&id=14982844.

[61] Folkman J: **Angiogenesis in cancer, vascular, rheumatoid and other disease.** *Nat Med* 1995, **1**:27–31, http://eutils.ncbi.nlm.nih.gov/entrez/eutils/elink.fcgi?cmd=prlinks\&db\%from=pubmed\&retmode=ref\&id=7584949.

[62] Jones M, Sarfeh I, Tarnawski A: **Induction of in vitro angiogenesis in the endothelial-derived cell line, EA hy926, by ethanol is mediated through PKC and MAPK.** *Biochem Biophys Res Commun* 1998, **249**:118–23, http://eutils.ncbi.nlm.nih.gov/entrez/eutils/elink.fcgi?cmd=prlinks\&db\%from=pubmed\&retmode=ref\&id=9705842.

[63] Jones M, Itani R, Wang H, Tomikawa M, Sarfeh I, Szabo S, Tarnawski A: **Activation of VEGF and Ras genes in gastric mucosa during angiogenic response to ethanol injury.** *Am J Physiol* 1999, **276**(6 Pt 1):G1345–55, http://eutils.ncbi.nlm.nih.gov/entrez/eutils/elink.fcgi?cmd=prlinks\&db\%from=pubmed\&retmode=ref\&id=10362637.

[64] Gavin T, Wagner P: **Acute ethanol increases angiogenic growth factor gene expression in rat skeletal muscle.** *J Appl Physiol* 2002, **92**(3):1176–82, http://eutils.ncbi.nlm.nih.gov/entrez/eutils/elink.fcgi?cmd=prlinks\&db\%from=pubmed\&retmode=ref\&id=11842056.

[65] Black R: **TIMP3 checks inflammation.** *Nat Genet* 2004, **36**(9):934–5, http://eutils.ncbi.nlm.nih.gov/entrez/eutils/elink.fcgi?cmd=prlinks\&db\%from=pubmed\&retmode=ref\&id=15340428.

[66] Muhs B, Patel S, Yee H, Marcus S, Shamamian P: **Inhibition of matrix metalloproteinases reduces local and distant organ injury following experimental acute pancreatitis.** *J Surg Res* 2003, **109**(2):110–7, http://eutils.ncbi.nlm.nih.gov/entrez/eutils/elink.fcgi?cmd=prlinks\&db\%from=pubmed\&retmode=ref\&id=12643851.

[67] Nakae H, Endo S, Inoue Y, Fujino Y, Wakabayashi G, Inada K, Sato S: **Matrix metalloproteinase-1 and cytokines in patients with acute pancreatitis.** *Pancreas* 2003, **26**(2):134–8, http://eutils.ncbi.nlm.nih.gov/entrez/eutils/elink.fcgi?cmd=prlinks\&db\%from=pubmed\&retmode=ref\&id=12604910.

[68] Phillips P, McCarroll J, Park S, Wu M, Pirola R, Korsten M, Wilson J, Apte M: **Rat pancreatic stellate cells secrete matrix metalloproteinases: implications for extracellular matrix turnover.** *Gut* 2003, **52**(2):275–82, http://eutils.ncbi.nlm.nih.gov/entrez/eutils/elink.fcgi?cmd=prlinks\&db\%from=pubmed\&retmode=ref\&id=12524413.

[69] Bramhall S, Neoptolemos J, Stamp G, Lemoine N: **Imbalance of expression of matrix metalloproteinases (MMPs) and tissue inhibitors of the matrix metalloproteinases (TIMPs) in human pancreatic carcinoma.** *J Pathol* 1997, **182**(3):347–55, http://eutils.ncbi.nlm.nih.gov/entrez/eutils/elink.fcgi?cmd=prlinks\&db\%from=pubmed\&retmode=ref\&id=9349239.

[70] Bhatnagar A, Wig J, Majumdar S: **Immunological findings in acute and chronic pancreatitis.** *ANZ J Surg* 2003, **73**(1-2):59–64, http://eutils.ncbi.nlm.nih.gov/entrez/eutils/elink.fcgi?cmd=prlinks\&db\%from=pubmed\&retmode=ref\&id=12534743.

[71] Sacchi N, Watson D, Guerts van Kessel A, Hagemeijer A, Kersey J, Drabkin H, Patterson D, Papas T: **Hu-ets-1 and Hu-ets-2 genes are transposed in acute leukemias with (4;11) and (8;21) translocations.** *Science* 1986, **231**(4736):379–82, http://eutils.ncbi.nlm.nih.gov/entrez/eutils/elink.fcgi?cmd=prlinks\&db\%from=pubmed\&retmode=ref\&id=3941901.

[72] Qian X, Rothman V, Nicosia R, Tuszynski G: **Expression of thrombospondin-1 in human pancreatic adenocarcinomas: role in matrix metalloproteinase-9 production.** *Pathol Oncol Res* 2001, **7**(4):251–9, http://eutils.ncbi.nlm.nih.gov/entrez/eutils/elink.fcgi?cmd=prlinks\&db\%from=pubmed\&retmode=ref\&id=11882904.

[73] Tobita K, Kijima H, Dowaki S, Oida Y, Kashiwagi H, Ishii M, Sugio Y, Sekka T, Ohtani Y, Tanaka M, Inokuchi S, Makuuchi H: **Thrombospondin-1 expression as a prognostic predictor of pancreatic ductal carcinoma.** *Int J Oncol* 2002, **21**(6):1189–95, http://eutils.ncbi.nlm.nih.gov/entrez/eutils/elink.fcgi?cmd=prlinks\&db\%from=pubmed\&retmode=ref\&id=12429967.

[74] Hu Y, Komorowski RA, Graewin S, Hostetter G, Kallioniemi O, Pitt H, Ahrendt S: **Thymidylate synthase expression predicts the response to 5-fluorouracil-based adjuvant therapy in pancreatic cancer.** *Clin Cancer Res* 2003, **9**(11):4165–71.

[75] Nakamori S, Ishikawa O, Ohhigashi H, Kameyama M, Furukawa H, Sasaki Y, Inaji H, Higashiyama M, Imaoka S, Iwanaga T, et al: **Expression of nucleoside diphosphate kinase/nm23 gene product in human pancreatic cancer: an association with lymph node metastasis and tumor invasion.** *Clin Exp Metastasis* 1993, **11**(2):151–8, http://eutils.ncbi.nlm.nih.gov/entrez/eutils/elink.fcgi?cmd=prlinks\&db\%from=pubmed\&retmode=ref\&id=8383029.

[76] Unoki M, Nakamura Y: **EGR2 induces apoptosis in various cancer cell lines by direct transactivation of BNIP3L and BAK.** *Oncogene* 2003, **22**(14):2172–85, http://eutils.ncbi.nlm.nih.gov/entrez/eutils/elink.fcgi?cmd=prlinks\&db\%from=pubmed\&retmode=ref\&id=12687019.

[77] Unoki M, Nakamura Y: **Growth-suppressive effects of BPOZ and EGR2, two genes involved in the PTEN signaling pathway.** *Oncogene* 2001, **20**(33):4457–65, http://eutils.ncbi.nlm.nih.gov/entrez/eutils/elink.fcgi?cmd=prlinks\&db\%from=pubmed\&retmode=ref\&id=11494141.

[78] Smith M, Wilson M, Hamanaka R, Chase D, Kung H, Longo D, Ferris D: **Malignant transformation of mammalian cells initiated by constitutive expression of the polo-like kinase.** *Biochem Biophys Res Commun* 1997, **234**(2):397–405, http://eutils.ncbi.nlm.nih.gov/entrez/eutils/elink.fcgi?cmd=prlinks\&db\%from=pubmed\&retmode=ref\&id=9177283.

[79] Liu X, Erikson R: **Polo-like kinase (Plk)1 depletion induces apoptosis in cancer cells.** *Proc Natl Acad Sci U S A* 2003, **100**(10):5789–94, http://eutils.ncbi.nlm.nih.gov/entrez/eutils/elink.fcgi?cmd=prlinks\&db\%from=pubmed\&retmode=ref\&id=12732729.

[80] Gray P Jr, Bearss D, Han H, Nagle R, Tsao M, Dean N, Von Hoff D: **Identification of human polo-like kinase 1 as a potential therapeutic target in pancreatic cancer.** *Mol Cancer Ther* 2004, **3**(5):641–6, http://eutils.ncbi.nlm.nih.gov/entrez/eutils/elink.fcgi?cmd=prlinks\&db\%from=pubmed\&retmode=ref\&id=15141022.

[81] Liu N, Bi F, Pan Y, Sun L, Xue Y, Shi Y, Yao X, Zheng Y, Fan D: **Reversal of the malignant phenotype of gastric cancer cells by inhibition of RhoA expression and activity.** *Clin Cancer Res* 2004, **10**(18 Pt 1):6239–47, http://eutils.ncbi.nlm.nih.gov/entrez/eutils/elink.fcgi?cmd=prlinks\&db\%from=pubmed\&retmode=ref\&id=15448013.

[82] Vasiliev J, Omelchenko T, Gelfand I, Feder H, Bonder E: **Rho overexpression leads to mitosis-associated detachment of cells from epithelial sheets: a link to the mechanism of cancer dissemination.** *Proc Natl Acad Sci U S A* 2004, **101**(34):12526–30, http://eutils.ncbi.nlm.nih.gov/entrez/eutils/elink.fcgi?cmd=prlinks\&db\%from=pubmed\&retmode=ref\&id=15304643.

[83] Kamai T, Yamanishi T, Shirataki H, Takagi K, Asami H, Ito Y, Yoshida K: **Overexpression of RhoA, Rac1, and Cdc42 GTPases is associated with progression in testicular cancer.** *Clin Cancer Res* 2004, **10**(14):4799–805, http://eutils.ncbi.nlm.nih.gov/entrez/eutils/elink.fcgi?cmd=prlinks\&db\%from=pubmed\&retmode=ref\&id=15269155.

[84] Keyse S, Emslie E: **Oxidative stress and heat shock induce a human gene encoding a protein-tyrosine phosphatase.** *Nature* 1992, **359**(6396):644–7, http://eutils.ncbi.nlm.nih.gov/entrez/eutils/elink.fcgi?cmd=prlinks\&db\%from=pubmed\&retmode=ref\&id=1406996.

[85] Alessi D, Smythe C, Keyse S: **The human CL100 gene encodes a Tyr/Thr-protein phosphatase which potently and specifically inactivates MAP kinase and suppresses its activation by oncogenic ras in Xenopus oocyte extracts.** *Oncogene* 1993, **8**(7):2015–20,

http://eutils.ncbi.nlm.nih.gov/entrez/eutils/elink.fcgi?cmd=prlinks\&db\%from=pubmed\&retmode=ref\&id=8390041.

[86] Beißbarth T, Fellenberg K, Brors B, Arribas-Prat R, Boer J, Hauser N, Scheideler M, Hoheisel J, Schutz G, Poustka A, Vingron M: **Processing and quality control of DNA array hybridization data.** *Bioinformatics* 2000, **16**(11):1014–22, http://eutils.ncbi.nlm.nih.gov/entrez/eutils/elink.fcgi?cmd=prlinks\&db\%from=pubmed\&retmode=ref\&id=11159313.

[87] Fellenberg K, Hauser N, Brors B, Neutzner A, Hoheisel J, Vingron M: **Correspondence analysis applied to microarray data.** *Proc Natl Acad Sci U S A* 2001, **98**(19):10781–6, http://eutils.ncbi.nlm.nih.gov/entrez/eutils/elink.fcgi?cmd=prlinks\&db\%from=pubmed\&retmode=ref\&id=11535808.

[88] König R, Baldessari D, Pollet N, Niehrs C, Eils R: **Reliability of gene expression ratios for cDNA microarrays in multiconditional experiments with a reference design.** *Nucleic Acids Res* 2004, **32**(3):e29, http://eutils.ncbi.nlm.nih.gov/entrez/eutils/elink.fcgi?cmd=prlinks\&db\%from=pubmed\&retmode=ref\&id=14966261.

[89] Rousseeuw P: **Silhouettes: a graphical aid to the interpretation and validation of cluster analysis.** *Journal of Computational and Applied Mathematics* 1987, **20**:53–65.

[90] **SAM v1.21 - Excel macro** http://www-stat.stanford.edu/~tibs/clickwrap/sam/academic.

Figure 6

Further analysis of cluster 4 (overview). The trait cluster of highest malignancy from Figs. 2 and 3 (green) has again been subdevided into four pieces by cutting the hierarchical clustering tree (panel c) at less than 20% of the variance within cluster four. The distribution of measurements across the traits is represented by percentages and by line thickness in panel c as for Fig. 2, and described in absolute numbers in the text.

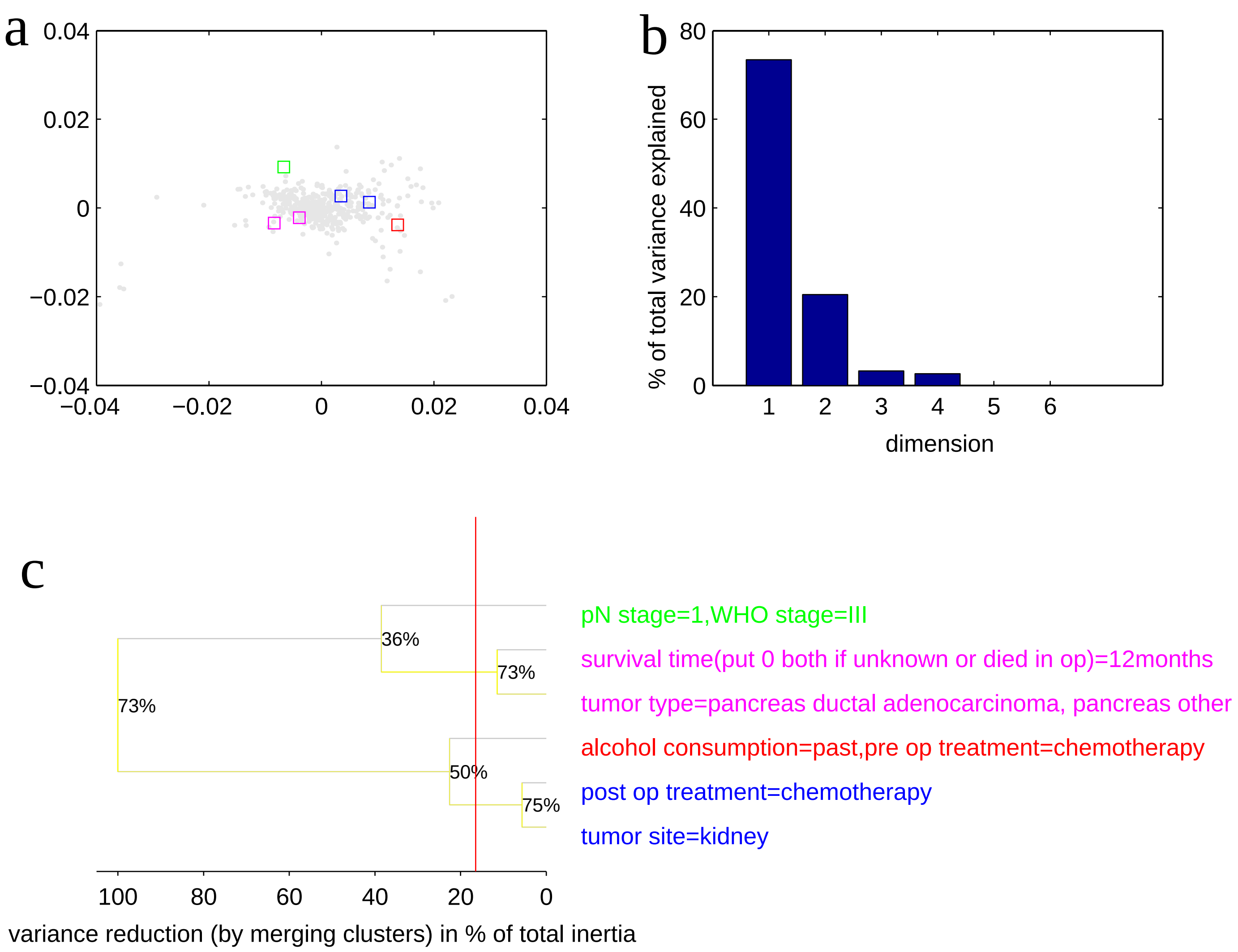

Figure 7

Trait-cluster ranges of cluster 4. The cluster centroids of Fig. 6 have been projected by CA, the first two axes explaining the entire variance among these (upper right). Layout and representation of objects follow Fig. 3, but for the representation of the measurements. Here, measurements annotated by at least one of the traits of cluster 4 are depicted as black, all others as grey boxes.

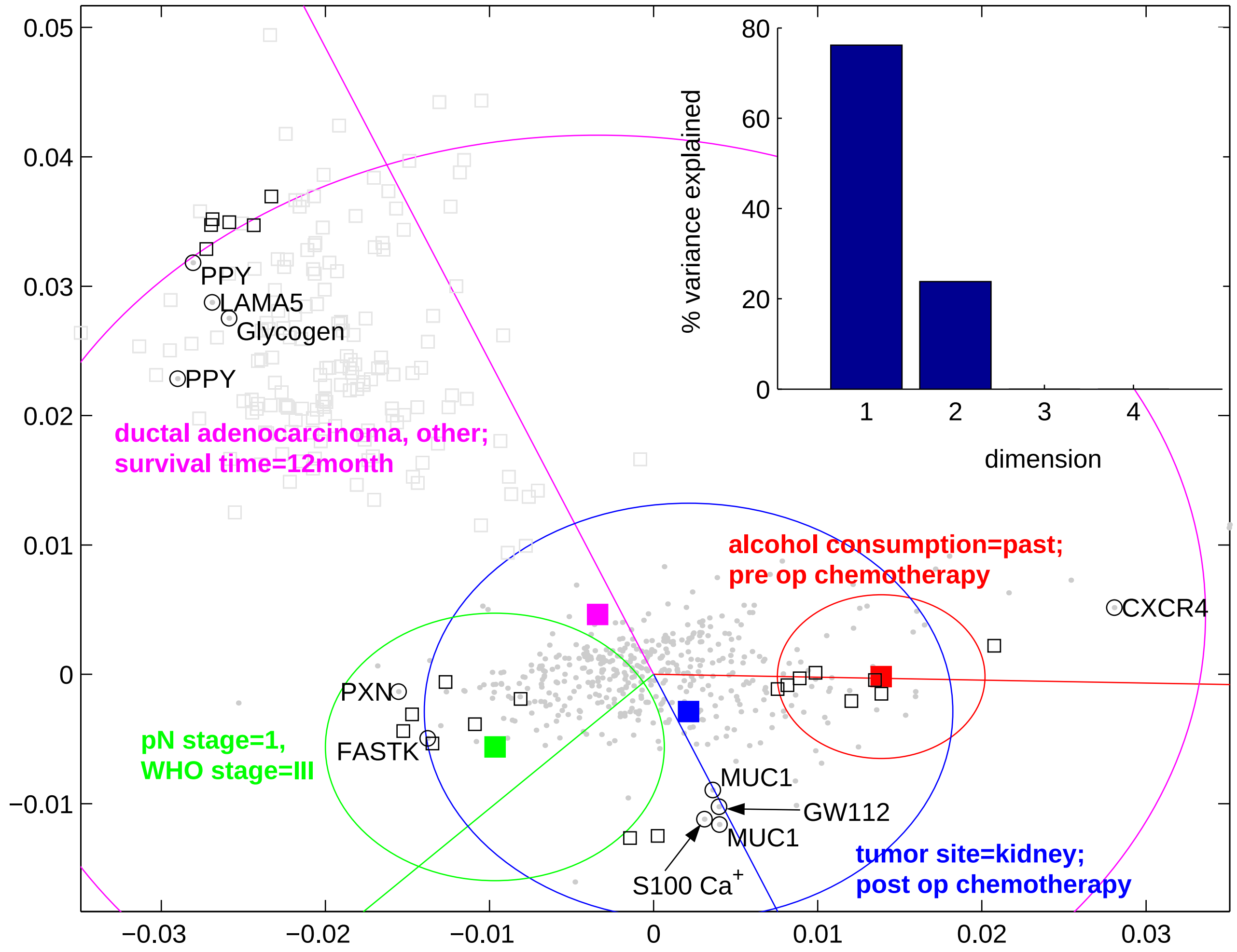

Figure 8a

Discretization decisions for the yeast data. Annotations `array individual', `total activity', `date of entry month', `experimentator hybridization', and `temperature' are shown by CA as well as by hierarchical clustering. The discretization was done by cutting a hierarchical tree at a particular level (vertical line).

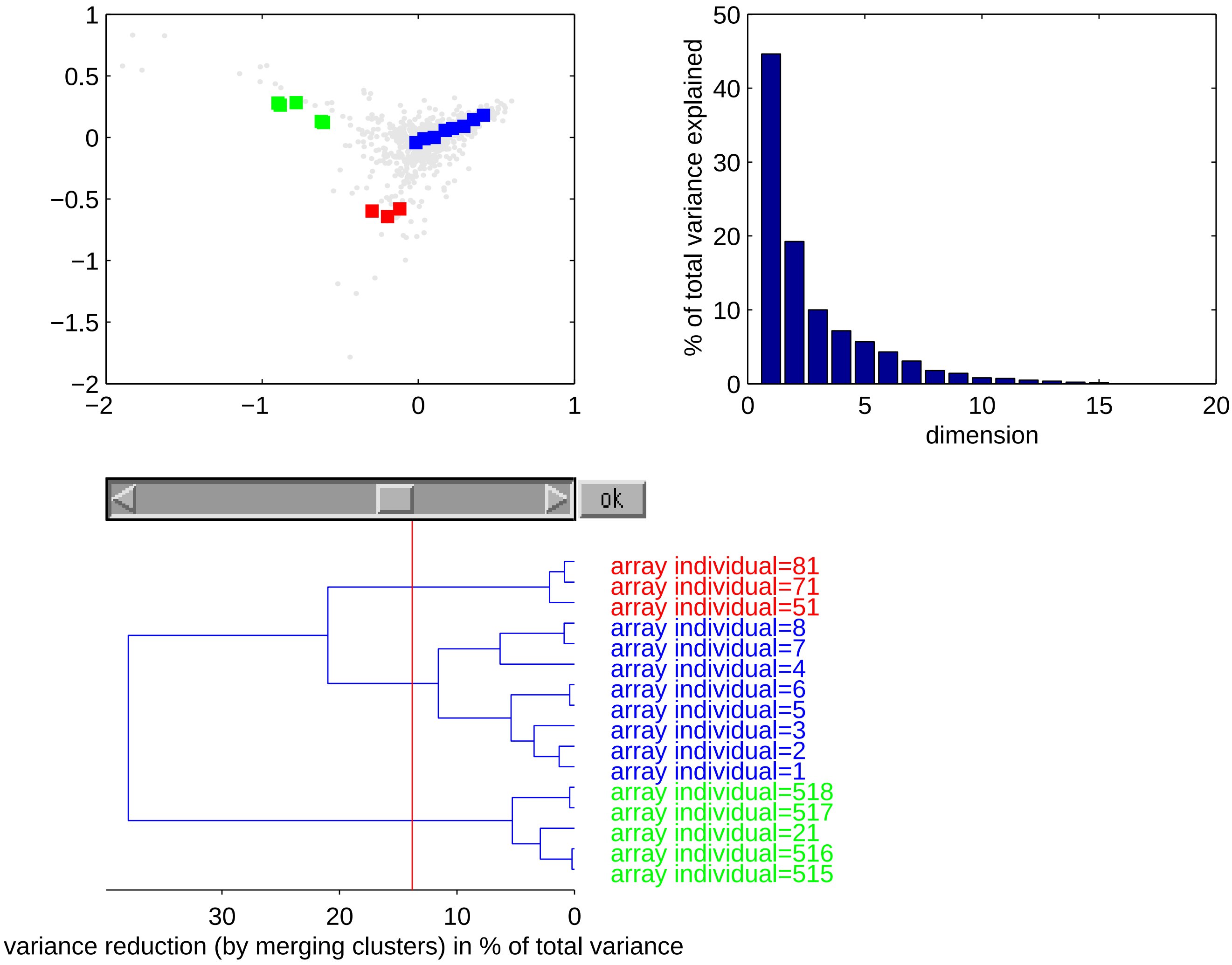

Figure 8b

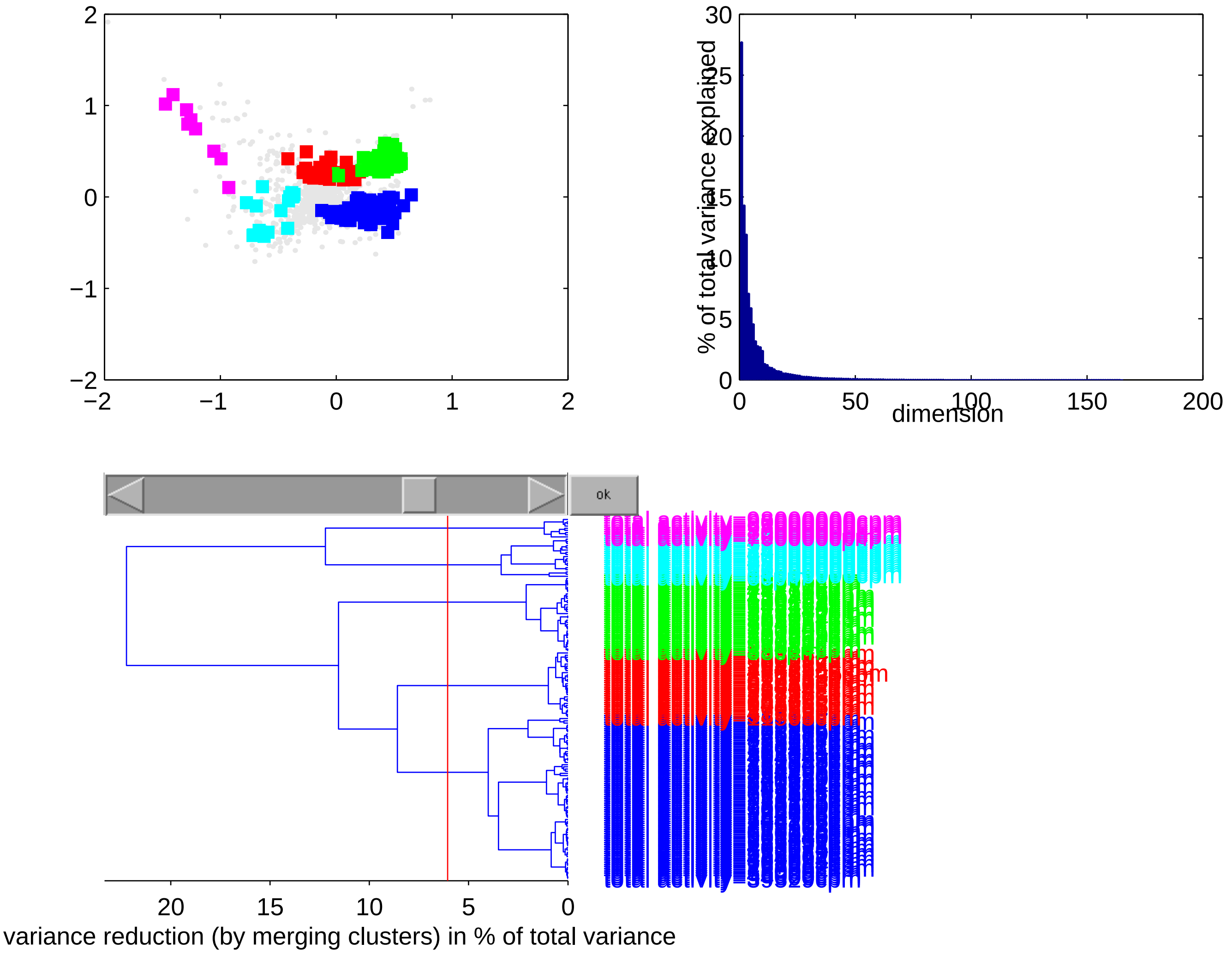

2
1
0
−1
−2
−2
−1
0
1
2
30
25
20
15
10
5
0
% of total variance explained
0
50
100
150
200
dimension
ok
20
15
10
5
0
variance reduction (by merging clusters) in % of total variance

Figure 8c

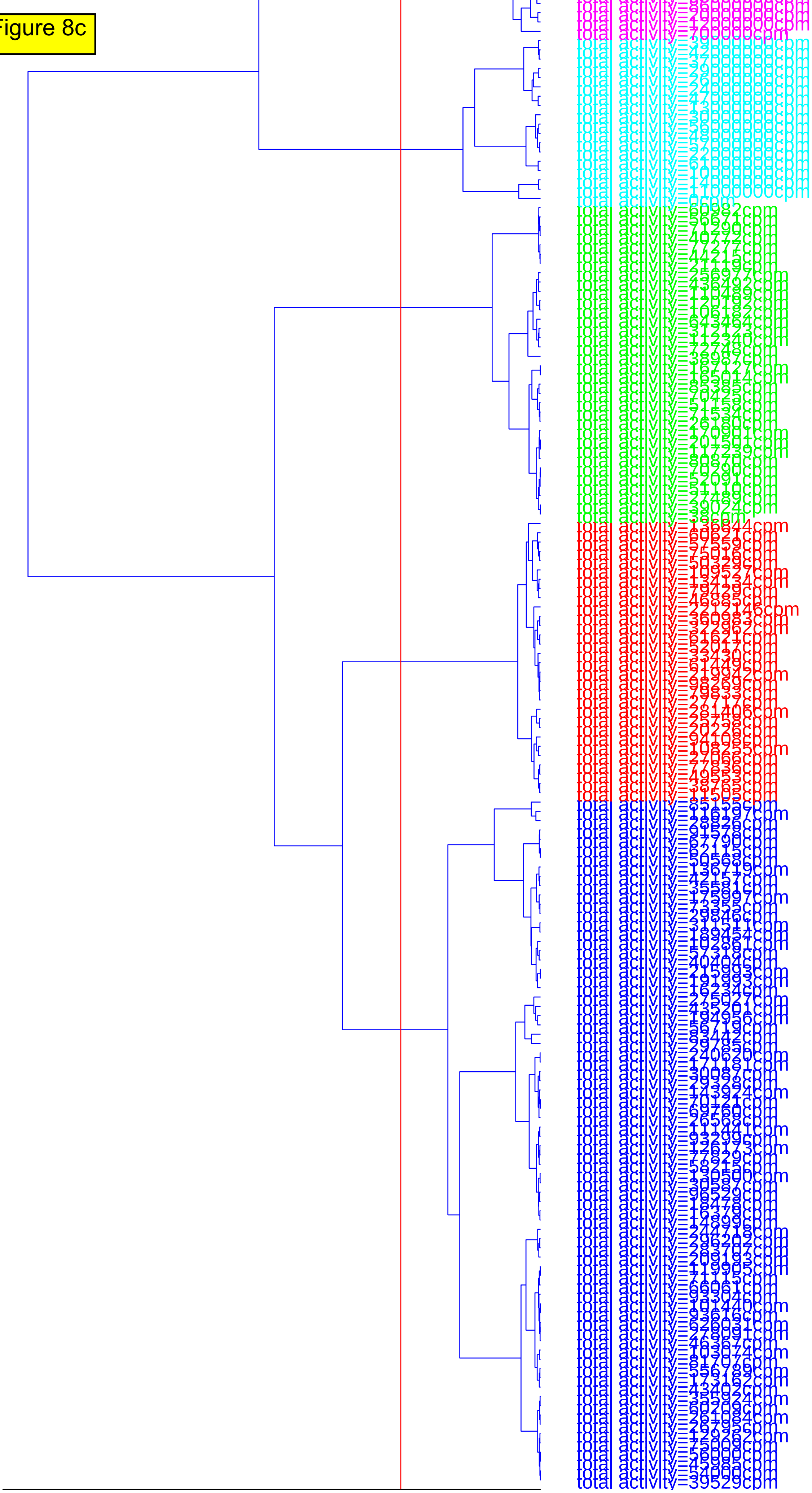

Figure 8d

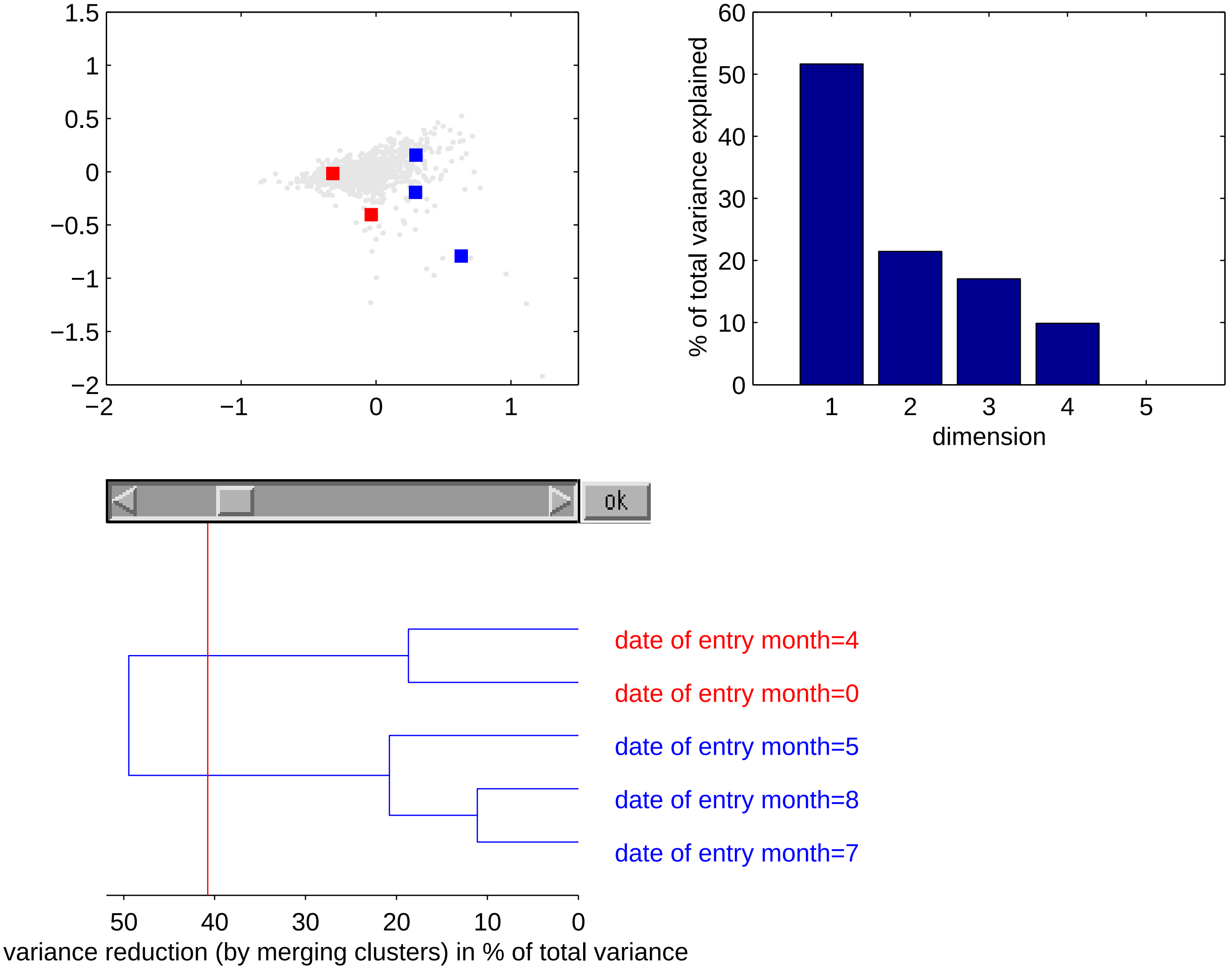

1.5
1
0.5
0
−0.5
−1
−1.5
−2
−2
−1
0
1
60
50
40
30
20
10
0
% of total variance explained
1
2
3
4
5
dimension
ok
date of entry month=4
date of entry month=0
date of entry month=5
date of entry month=8
date of entry month=7
50
40
30
20
10
0
variance reduction (by merging clusters) in % of total variance

Figure 8e

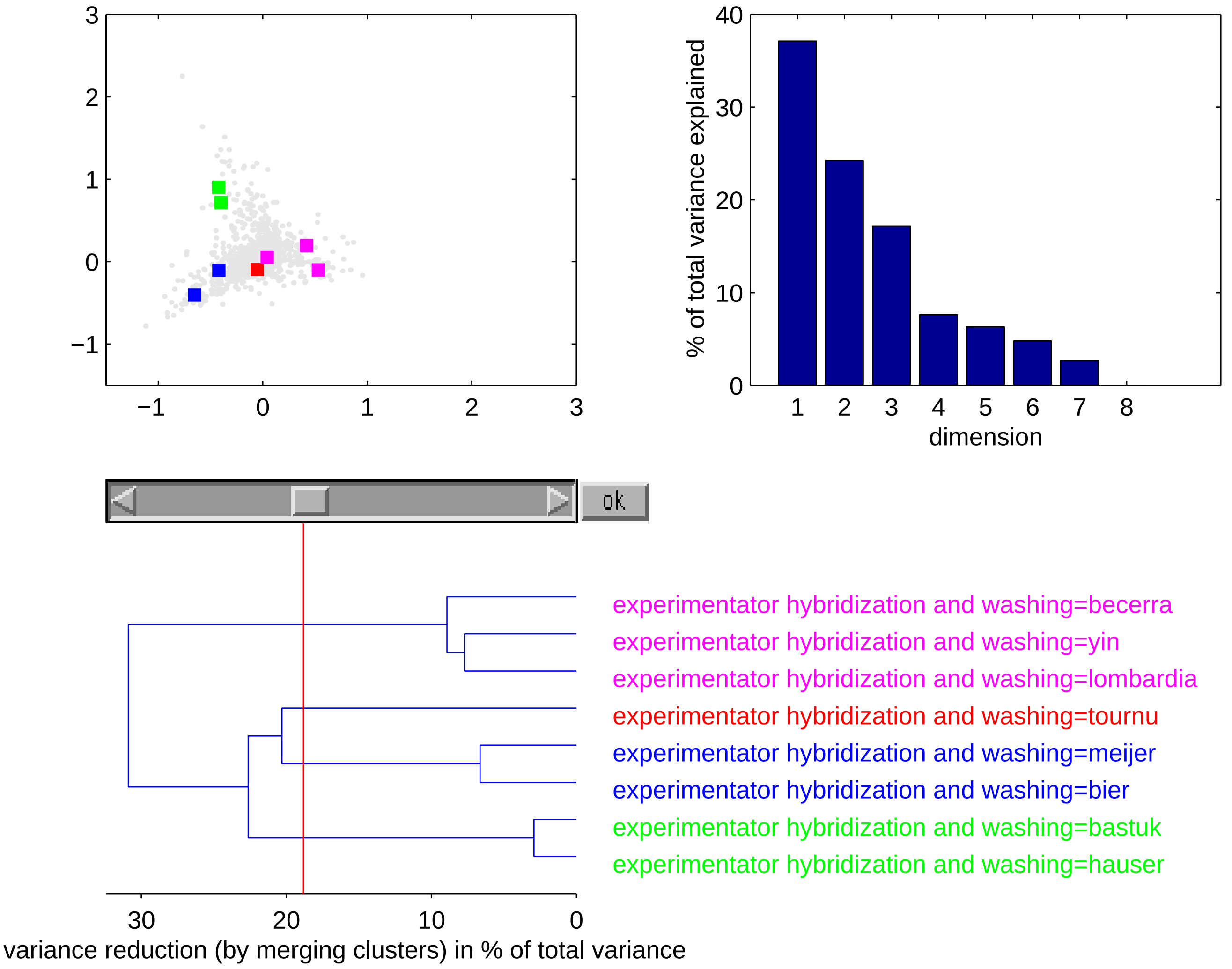
3
2
1
0
−1
−1
0
1
2
3
40
30
20
10
0
% of total variance explained
1
2
3
4
5
6
7
8
dimension
ok
experimentator hybridization and washing=becerra
experimentator hybridization and washing=yin
experimentator hybridization and washing=lombardia
experimentator hybridization and washing=tournu
experimentator hybridization and washing=meijer
experimentator hybridization and washing=bier
experimentator hybridization and washing=bastuk
experimentator hybridization and washing=hauser
30
20
10
0
variance reduction (by merging clusters) in % of total variance

Figure 8f

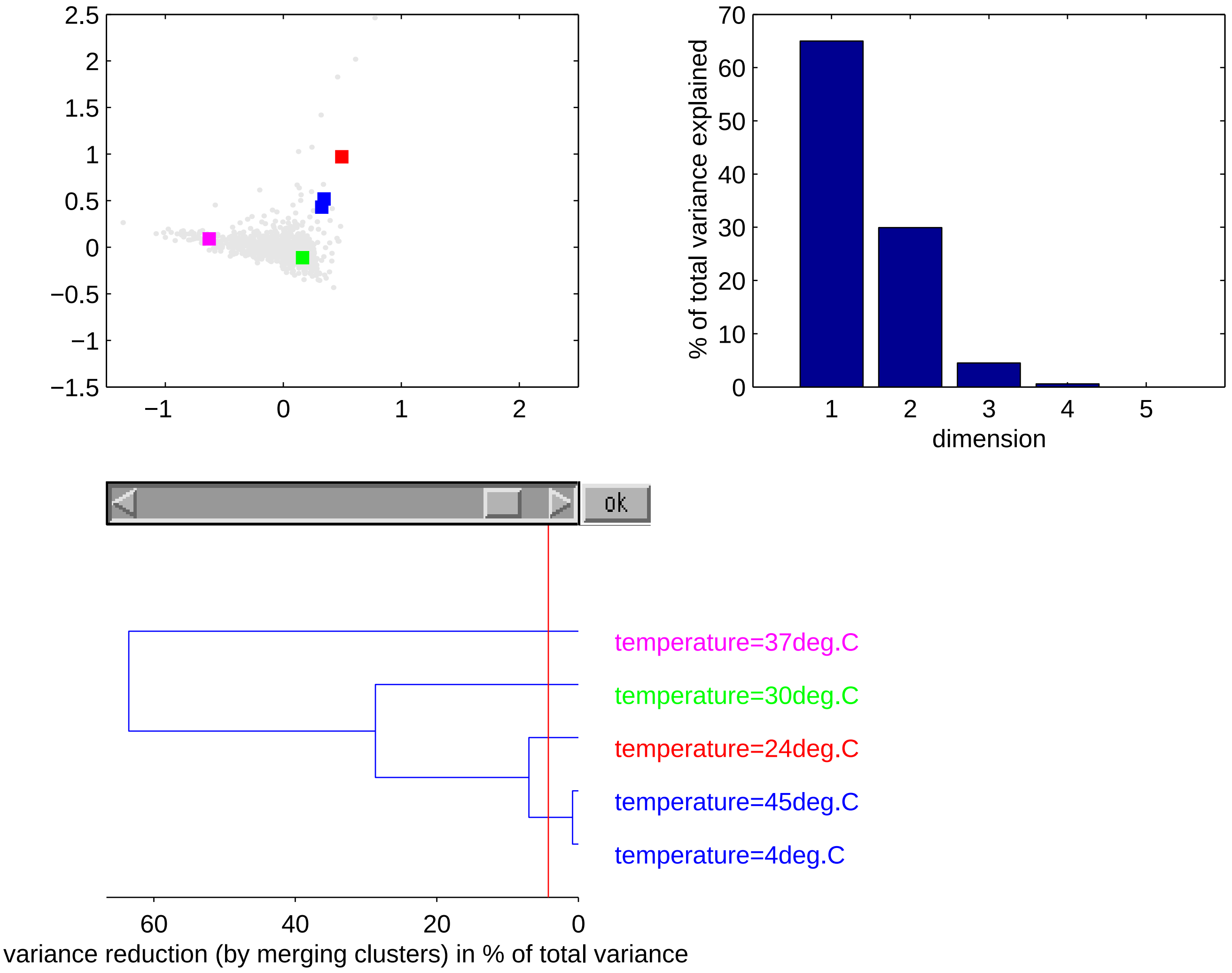

2.5
2
1.5
1
0.5
0
−0.5
−1
−1.5
−1
0
1
2
70
60
50
40
30
20
10
0
% of total variance explained
1
2
3
4
5
dimension
ok
temperature=37deg.C
temperature=30deg.C
temperature=24deg.C
temperature=45deg.C
temperature=4deg.C
60
40
20
0
variance reduction (by merging clusters) in % of total variance

Figure 9

Discretization decisions for the fly data. Annotation `array individual' is discretized by cutting the hierarchical tree at the level indicated by the vertical line.

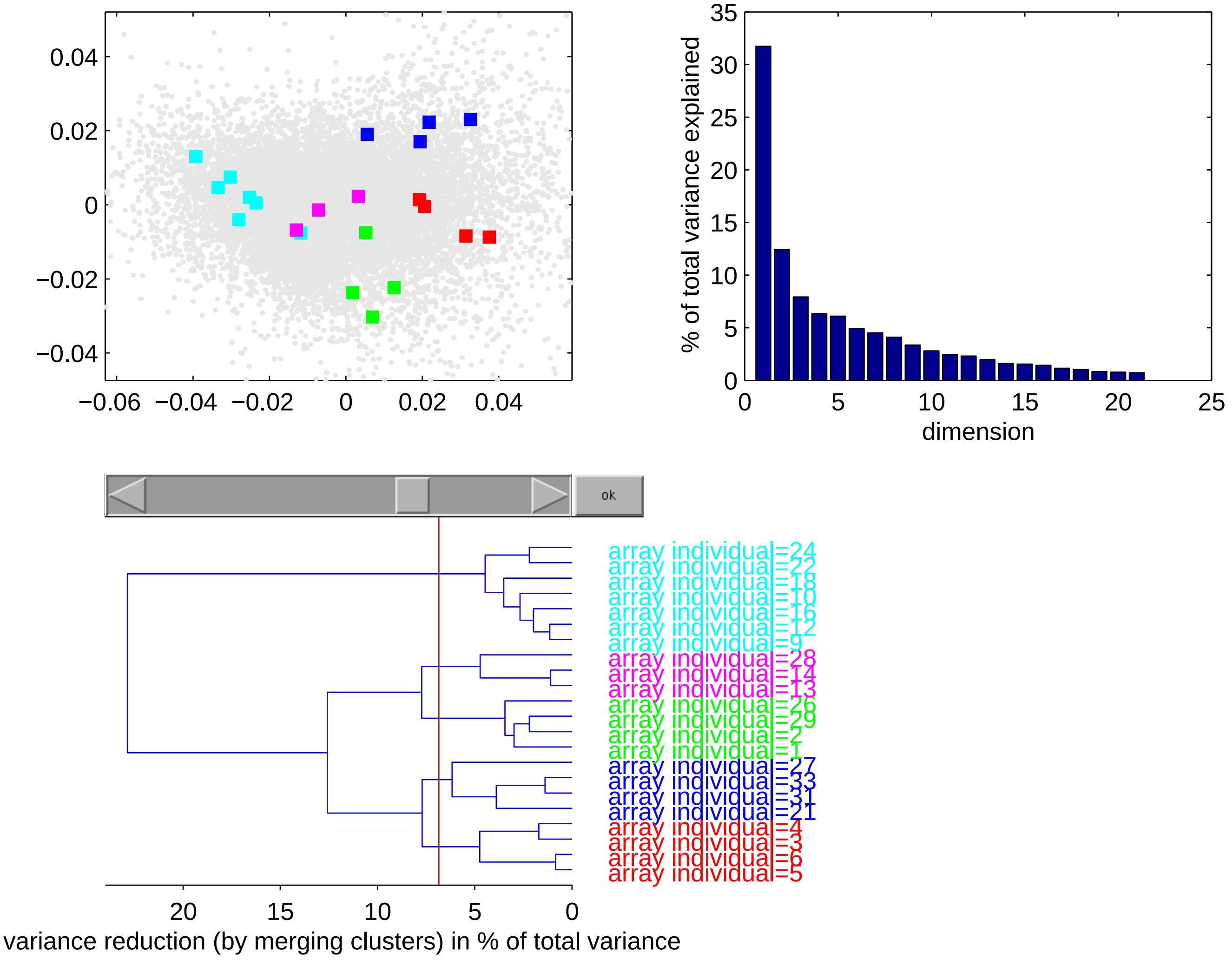

Figure 10a

Discretization decisions for the cancer data. Annotations `live status', `tumor type', `pT stage', `tumor subregion', `smoking', `alcohol consumption', `weight loss in last 4 weeks', and `OP procedure' are shown by CA as well as by hierarchical clustering. The red vertical line indicates the discretization level.

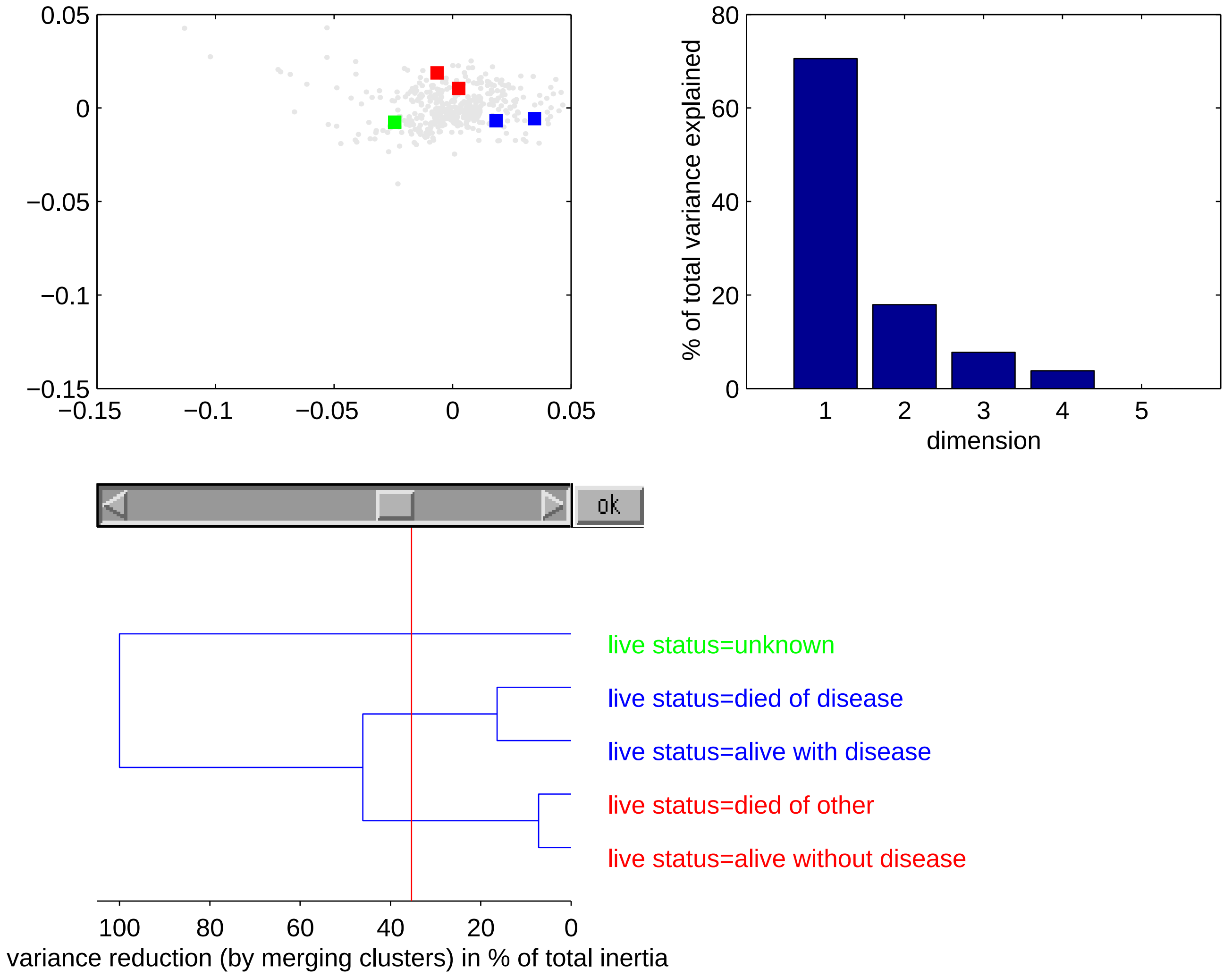

Figure 10b

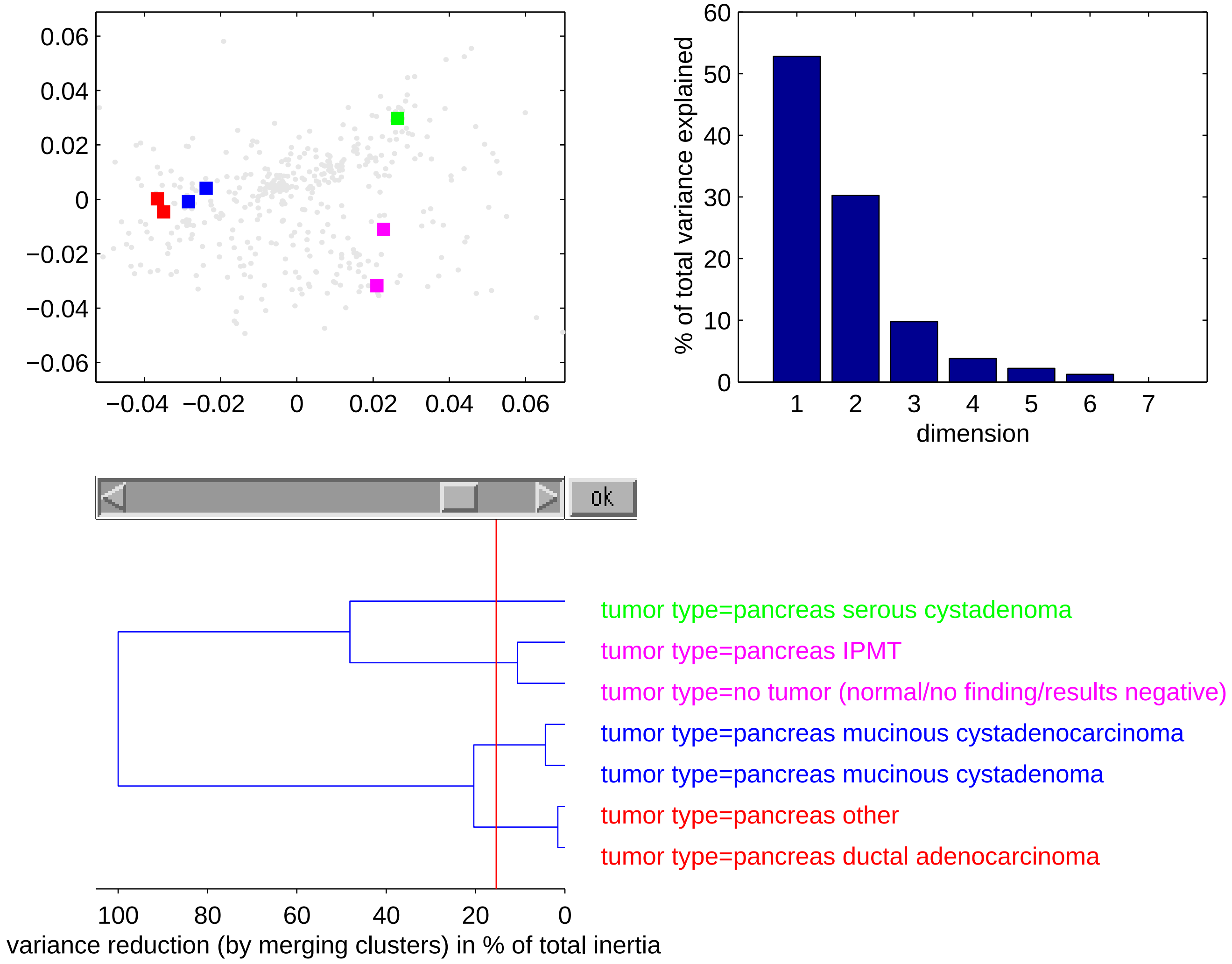

0.06
0.04
0.02
0
−0.02
−0.04
−0.06
−0.04 −0.02 0 0.02 0.04 0.06
60
50
40
30
20
10
0
% of total variance explained
1 2 3 4 5 6 7
dimension
ok
tumor type=pancreas serous cystadenoma
tumor type=pancreas IPMT
tumor type=no tumor (normal/no finding/results negative)
tumor type=pancreas mucinous cystadenocarcinoma
tumor type=pancreas mucinous cystadenoma
tumor type=pancreas other
tumor type=pancreas ductal adenocarcinoma
100 80 60 40 20 0
variance reduction (by merging clusters) in % of total inertia

Figure 10c

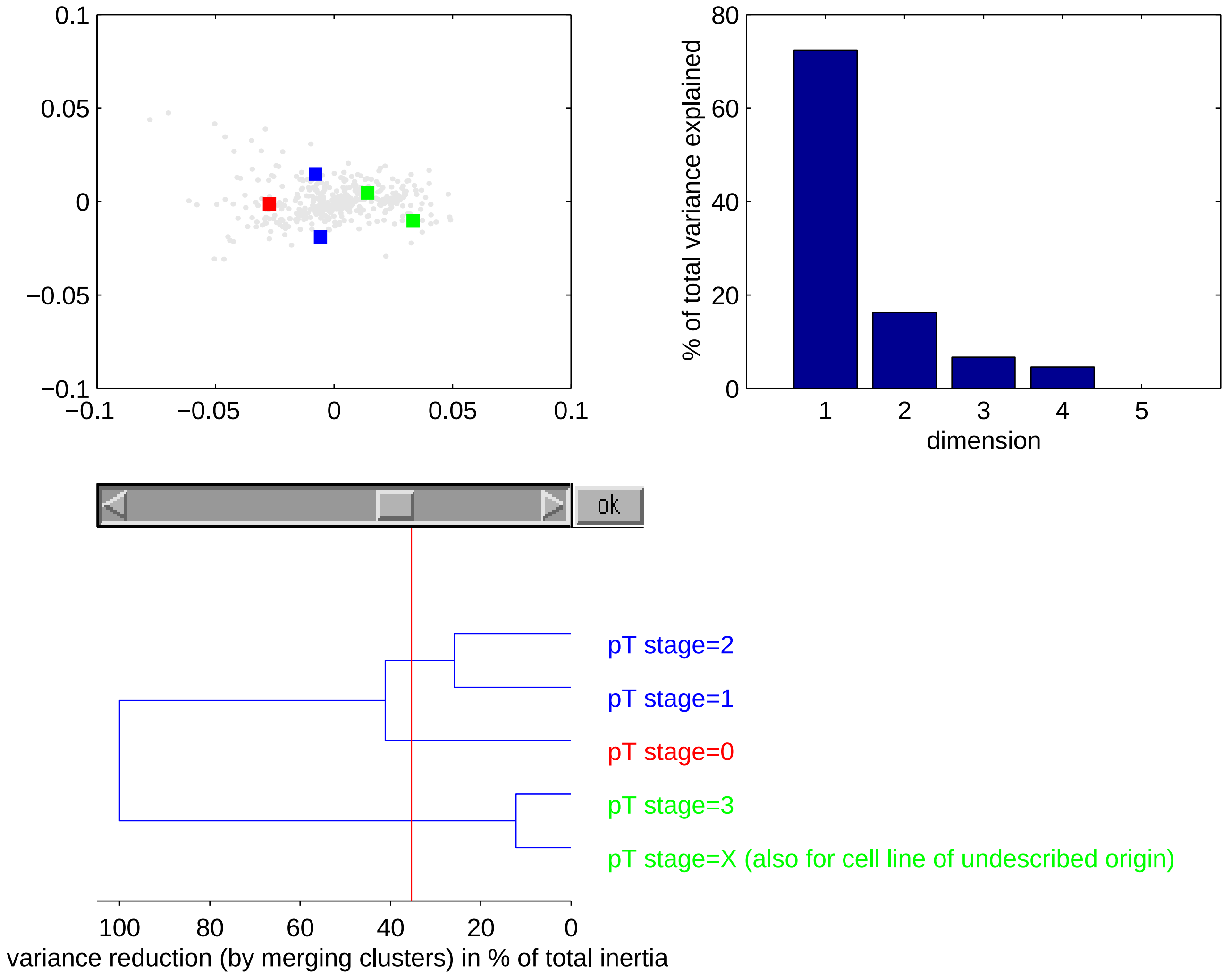
0.1
0.05
0
−0.05
−0.1
−0.1
−0.05
0
0.05
0.1
80
60
40
20
0
% of total variance explained
1
2
3
4
5
dimension
ok
pT stage=2
pT stage=1
pT stage=0
pT stage=3
pT stage=X (also for cell line of undescribed origin)
100
80
60
40
20
0
variance reduction (by merging clusters) in % of total inertia

Figure 10d

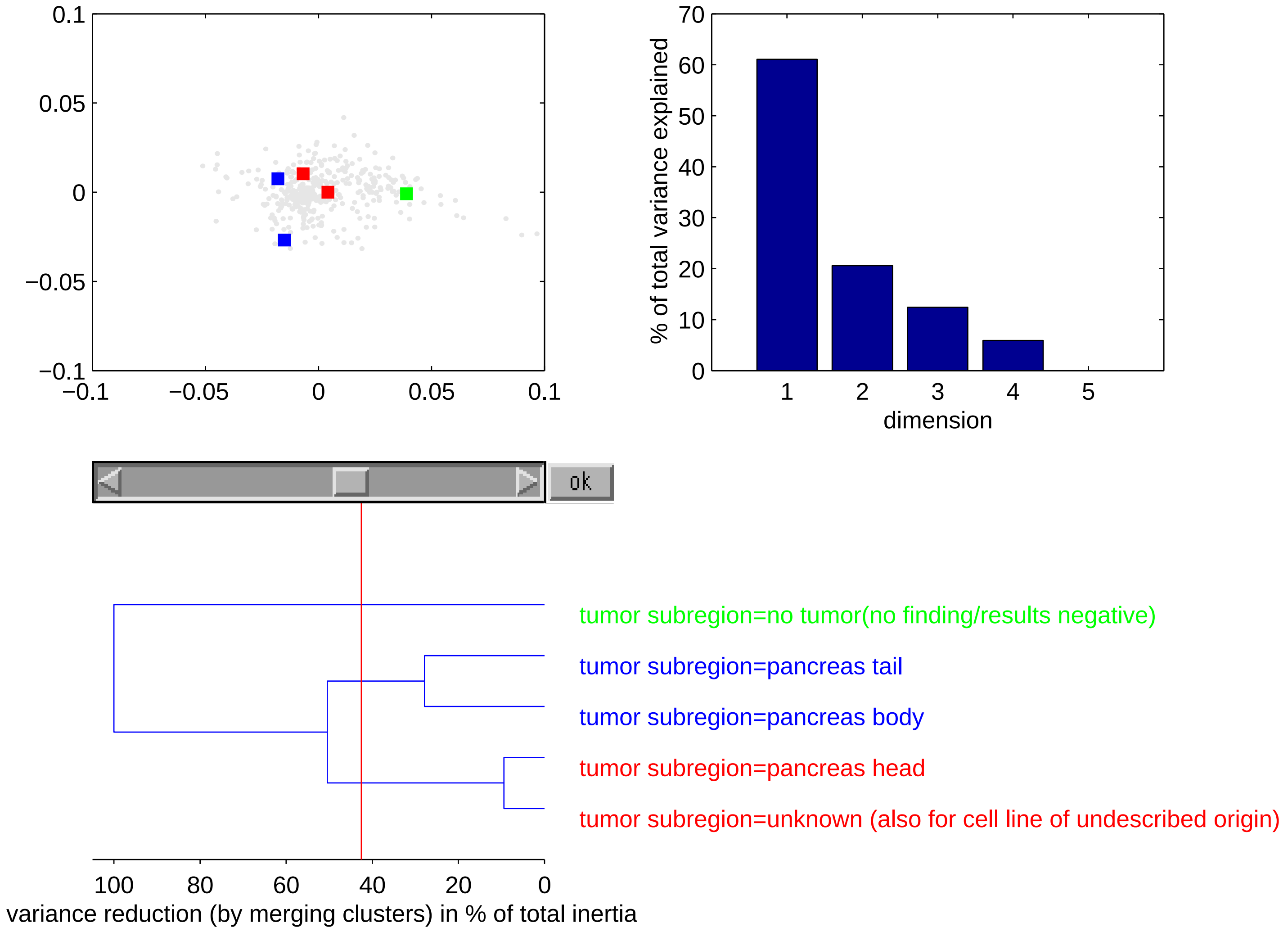
0.1
0.05
0
−0.05
−0.1
−0.1
−0.05
0
0.05
0.1
70
60
50
40
30
20
10
0
% of total variance explained
1
2
3
4
5
dimension
ok
tumor subregion=no tumor(no finding/results negative)
tumor subregion=pancreas tail
tumor subregion=pancreas body
tumor subregion=pancreas head
tumor subregion=unknown (also for cell line of undescribed origin)
100
80
60
40
20
0
variance reduction (by merging clusters) in % of total inertia

Figure 10e

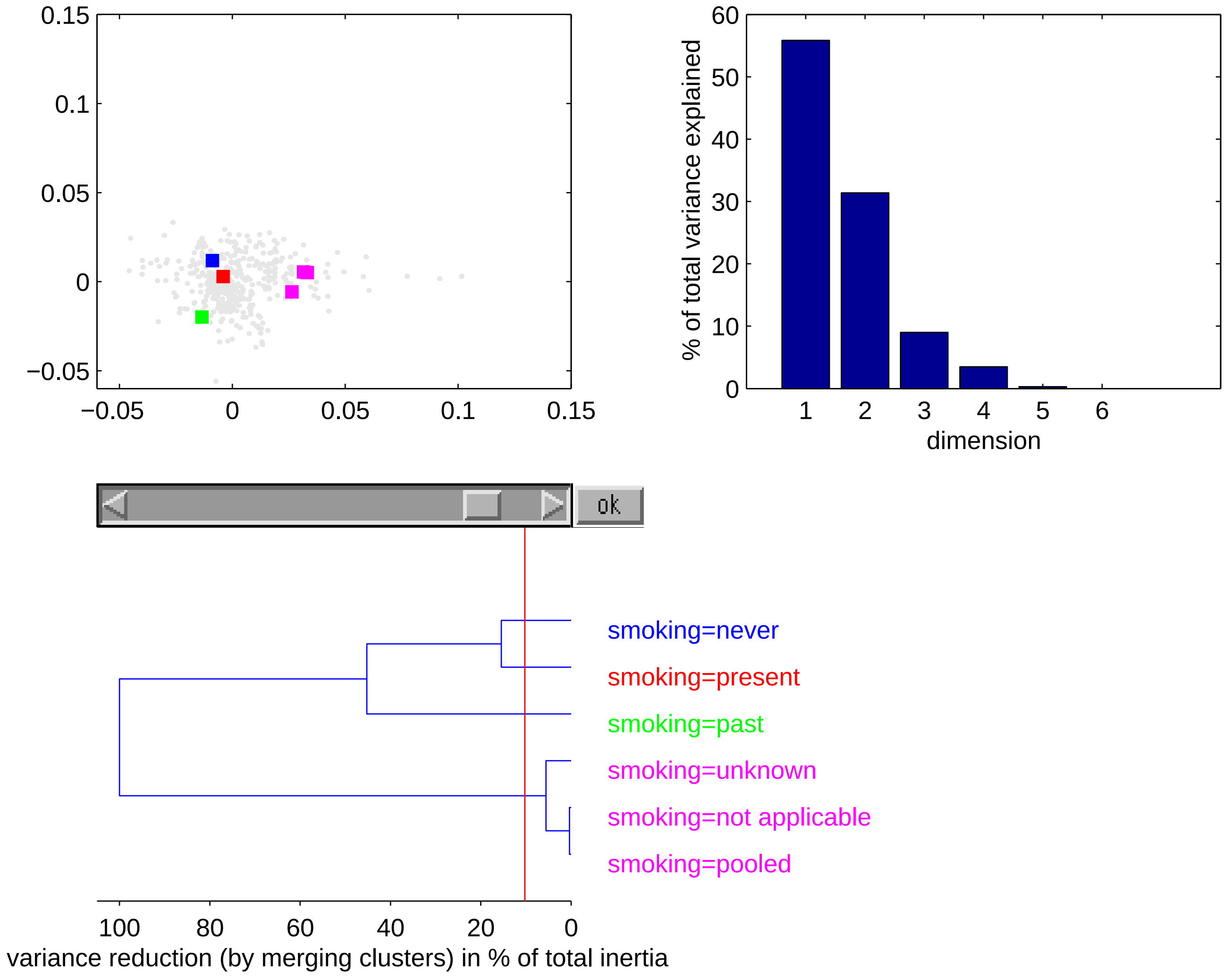

0.15
0.1
0.05
0
−0.05
−0.05
0
0.05
0.1
0.15
60
50
40
30
20
10
0
% of total variance explained
1
2
3
4
5
6
dimension
ok
smoking=never
smoking=present
smoking=past
smoking=unknown
smoking=not applicable
smoking=pooled
100
80
60
40
20
0
variance reduction (by merging clusters) in % of total inertia

Figure 10f

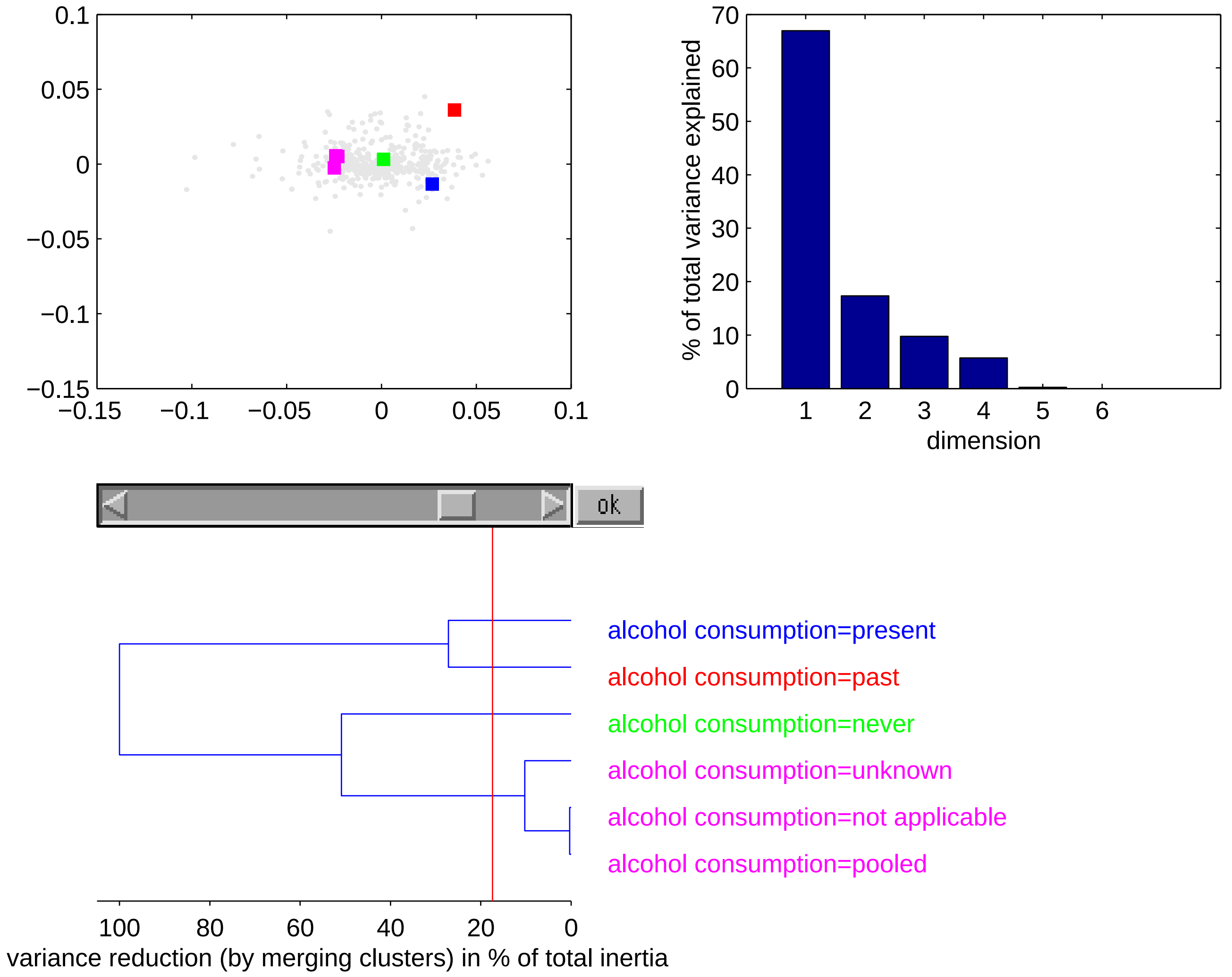
0.1
0.05
0
−0.05
−0.1
−0.15
−0.15
−0.1
−0.05
0
0.05
0.1
70
60
50
40
30
20
10
0
% of total variance explained
1
2
3
4
5
6
dimension
ok
alcohol consumption=present
alcohol consumption=past
alcohol consumption=never
alcohol consumption=unknown
alcohol consumption=not applicable
alcohol consumption=pooled
100
80
60
40
20
0
variance reduction (by merging clusters) in % of total inertia

Figure 10g

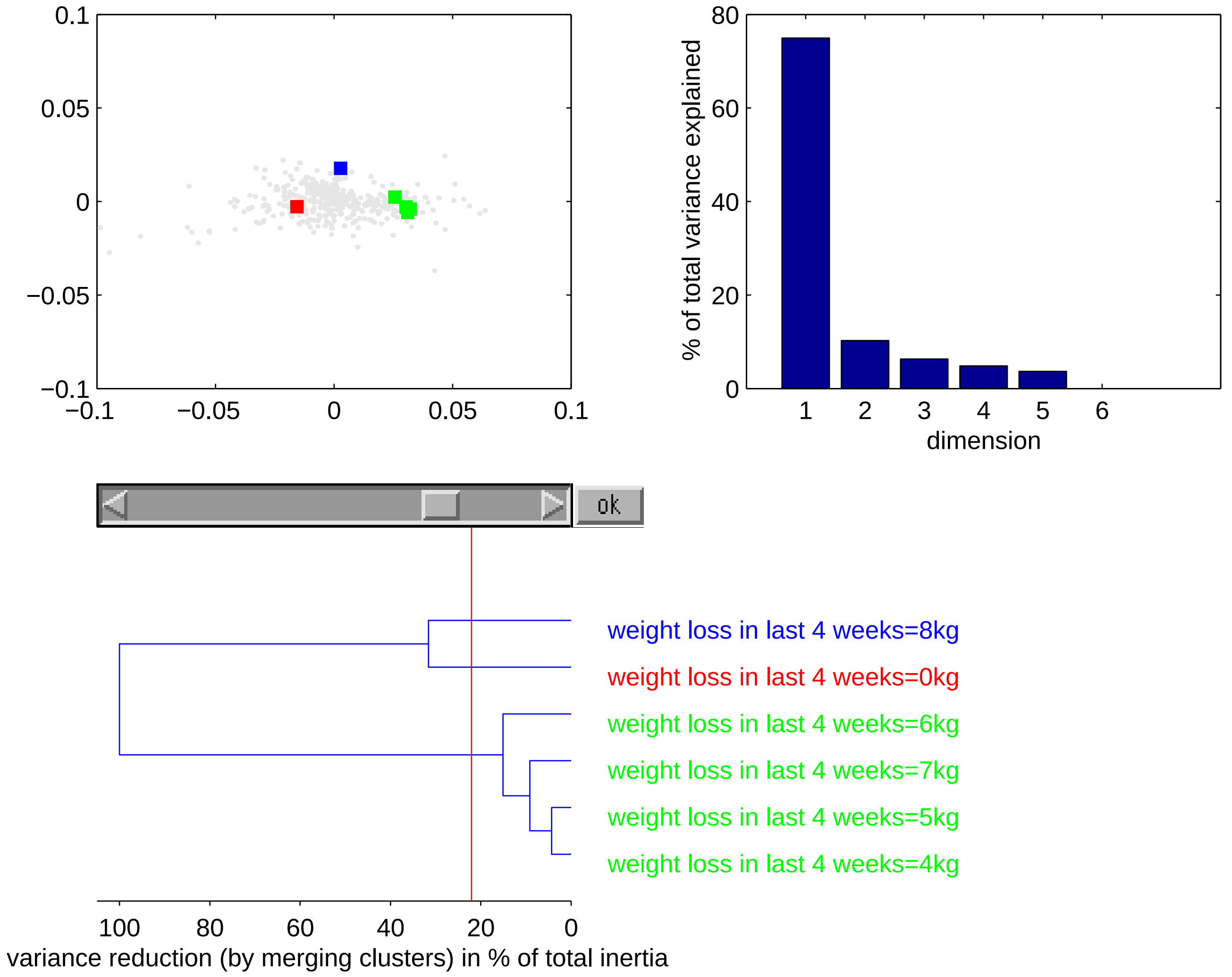

0.1
0.05
0
−0.05
−0.1
−0.1
−0.05
0
0.05
0.1
80
60
40
20
0
% of total variance explained
1
2
3
4
5
6
dimension
ok
weight loss in last 4 weeks=8kg
weight loss in last 4 weeks=0kg
weight loss in last 4 weeks=6kg
weight loss in last 4 weeks=7kg
weight loss in last 4 weeks=5kg
weight loss in last 4 weeks=4kg
100
80
60
40
20
0
variance reduction (by merging clusters) in % of total inertia

Figure 10h

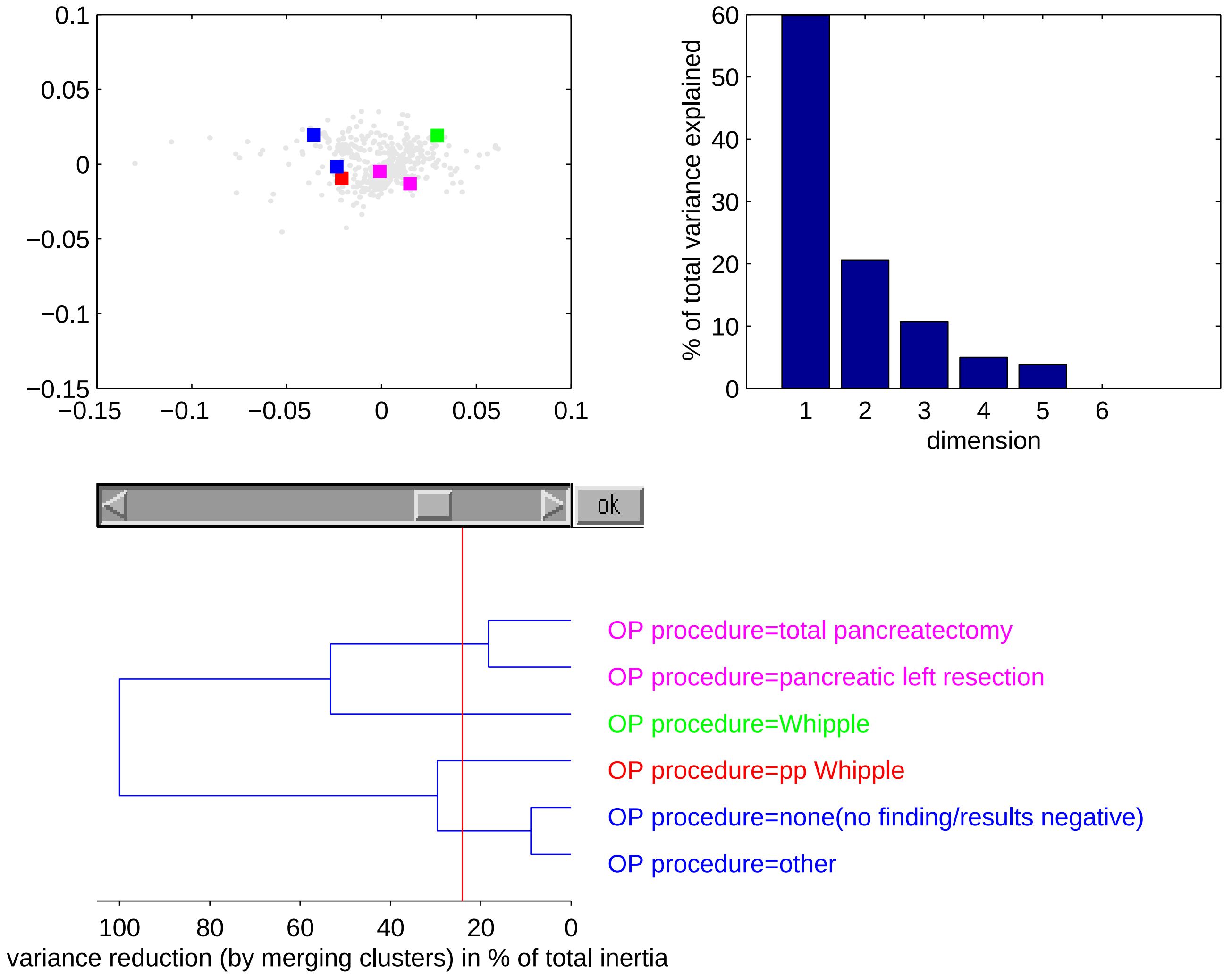
0.1
0.05
0
−0.05
−0.1
−0.15
−0.15
−0.1
−0.05
0
0.05
0.1
60
50
40
30
20
10
0
% of total variance explained
1
2
3
4
5
6
dimension
ok
OP procedure=total pancreatectomy
OP procedure=pancreatic left resection
OP procedure=Whipple
OP procedure=pp Whipple
OP procedure=none(no finding/results negative)
OP procedure=other
100
80
60
40
20
0
variance reduction (by merging clusters) in % of total inertia

Table 1: Eurofan annotations

Rank	χ^2 statistic	Inertia	Values	Annotation
1	657427967.7458	0.5398690615446	14	array_series
2	527634422.7475	0.4804637439625	8	total_activity
3	348495841.0354	0.3819823424118	11	transgene
4	326855640.6254	0.3600355782154	4	measuring_device
5	335481296.3458	0.3559206253439	8	strain
6	318619151.2093	0.3519742201367	4	experimentator_hybridization_and_washing
7	279386103.9804	0.303958008464	9	concentration
8	261862449.222	0.2892771753725	4	amount_of_RNA
9	253028626.1497	0.2785243002772	7	temporary_additive
10	222935985.7254	0.2501942962542	5	base
11	191915238.4296	0.2156767874388	3	intensity_measurement
12	190135578.0561	0.2118619451592	3	hybridisation_length
13	169005796.4711	0.1886484540309	4	temperature
14	143229382.6714	0.1594058795415	5	glucose
15	155735263.4378	0.1594017923315	3	array_individual
16	137427906.4269	0.1545766903104	3	mating_type
17	124406999.3193	0.1405994581241	3	buffer_volume
18	125647942.5163	0.1388190652014	4	amount_of_cDNA
19	115708589.6769	0.1300460711223	3	date_of_entry_year
20	112358318.8683	0.1272247357041	2	days_from_calibration_date
21	112358318.8683	0.1272247357041	2	2nd_transgene
22	112358318.8683	0.1272247357041	2	3rd_transgene
23	112358318.8683	0.1272247357041	2	4th_transgene
24	112358318.8683	0.1272247357041	2	5th_transgene
25	109507203.5286	0.1248941129859	2	buffer
26	100180705.229	0.114188261636	3	genetic_variation
27	98177941.20173	0.1118038287166	2	material_source
28	91671635.32437	0.104652425161	2	wash_buffer
29	91671635.32437	0.104652425161	2	prehybridization_without_target
30	91711387.35303	0.1043033822586	2	times
31	91711387.35303	0.1043033822586	2	wash_length
32	89990144.93296	0.102601442366	2	ADE2
33	90225023.18463	0.09981267809985	2	array_support
34	86270593.36098	0.09835704064225	2	date_of_entry_month
35	81547924.54347	0.0931292824536	2	priming
36	65051863.11485	0.07365904167313	2	LYS2
37	60928374.56896	0.06944534297338	2	URA3
38	57995536.55707	0.06735205373868	2	sucrose
39	57995536.55707	0.06735205373868	2	culture
40	57411351.87018	0.06528110310183	2	energy_metabolism
41	56891496.286	0.06485335589975	2	LEU2
42	45690598.83318	0.05208491357839	2	TRP1
43	44281898.9524	0.05048260141709	3	growth_phase
44	36408547.24089	0.04149026651875	2	HIS3

Table 2: Drosophila annotations

Rank	χ^2 statistic	Inertia	Values	Annotation
1	2641.5609	0.0032744139	5	embryo
2	1678.1186	0.0020801545	4	pupa
3	1903.8325	0.0012422805	5	array_individual
4	1197.7867	0.0011089874	2	array_series
5	794.57169	0.00098493152	2	adult
6	280.75228	0.00034801362	2	larva
7	464.01456	0.0002860859	2	label

Table 3: Cancer annotations

Rank	χ^2 statistic	Inertia	Silhouettes	Values	Annotation
1	276.9226	0.001164068	0.2367084	4	tumor_type
2	143.4693	0.0006030856	0.2012822	4	OP_procedure
3	146.4905	0.0006006496	0.1503014	4	tumor_site
4	138.4549	0.0005677015	0.2727973	4	alcohol_consumption
5	135.6649	0.0005562618	0.1672197	4	post_op_treatment
6	120.4296	0.0004937932	0.1831729	4	pre_op_treatment
7	113.6428	0.0004659656	0.09768074	3	live_status
8	109.3597	0.0004597032	0.2168474	3	weight_loss_in_last_4_weeks
9	110.8875	0.000454668	0.1108903	4	smoking
10	114.372	0.0004270137	-0.1227595	5	array_series
11	100.0451	0.0004205484	0.2271336	3	weight_loss
12	99.36025	0.0004074032	0.127146	3	pT_stage
13	98.36448	0.0004033203	0.2083806	3	tumor_subregion
14	87.65164	0.0003593948	0.1068296	4	sex
15	85.99255	0.0003525921	0.124933	3	race
16	83.29947	0.0003501566	0.1625618	5	tumor_size
17	77.51741	0.0003258512	0.38595	2	tumor_or_normal
18	77.51741	0.0003258512	0.38595	2	tumor
19	77.51741	0.0003258512	0.38595	2	differentiation_grade
20	77.51741	0.0003258512	0.38595	2	stroma
21	76.6868	0.0003144361	0.1879274	3	pN_stage
22	75.93271	0.0003038749	-0.01203282	3	previous_malignancy
23	70.99776	0.0002984453	0.3122639	3	survival_time
24	69.07486	0.0002903622	0.1920123	5	WHO_stage
25	67.59287	0.0002771486	0.08395655	2	percentage_normal
26	67.59287	0.0002771486	0.08395655	2	normal
27	63.66533	0.0002676228	0.2854392	2	pain
28	59.4302	0.0002436795	0.2390919	3	metastasis_location
29	53.63657	0.000214648	-0.06701474	3	OP_date_day
30	53.63657	0.000214648	-0.06701474	3	OP_date_day_month
31	53.63657	0.000214648	-0.06701474	3	OP_date_day_year
32	38.60706	0.0001582991	0.1140807	2	pM_stage
33	37.32228	0.0001493599	0.1386609	3	birth_date
34	32.38756	0.000136144	0.2813401	3	Diabetes_mellitus
35	31.0763	0.000127421	0.08725372	2	resection_status
36	24.86353	0.000104516	0.3959583	2	pancreatitis_in_history
37	17.47987	7.347819e-05	0.406136	2	jaundice
38	15.06363	4.869222e-05	0.1902052	2	label
39	6.696245	2.745637e-05	0.03480075	2	PMT_setting
40	6.696245	2.745637e-05	0.03480075	2	laser_power_setting
41	5.320251	2.181443e-05	0.04330024	2	CCD_camera_exposure_time

Table 4: Alcohol consumption

Rank	Inertia	(in %)	Silhouettes	Measurements	Alcohol consumtion
1	0.0002085	(36.7%)	0.63786	218	pooled, unknown, not applicable
2	0.0001452	(25.6%)	0.51514	8	past
3	0.0001881	(33.1%)	0.18197	42	present
4	2.5903e-05	(4.6%)	-0.24379	80	never

Table 5: Cancer annotation values (sorted by Silhouette values)

Rank	Inertia contribution	(in %)	Silhouettes	Measurements	Annotation=annotation value
1	1.094e-06	(0.4%)	1	152	sex=pooled,race=pooled,birth date=pooled
2	7.9986e-06	(2.6%)	0.89057	202	tumor or normal=normal,tumor=none (normal/no finding/results negative),differentiation grade (of tumor biopsy or original tumor in case of cell line)=no tumor(no finding/results negative),stroma=no tumor(no finding/results negative)
3	3.7662e-06	(1.2%)	0.81693	170	post op treatment=no tumor resection(e.g. cell line or biopsy of normal tissue),pre op treatment=no tumor resection(e.g. cell line or biopsy of normal tissue),metastasis location=none(no finding/results negative)
4	6.6447e-06	(2.1%)	0.76912	214	OP procedure=other, none(no finding/results negative)
5	9.4082e-06	(3%)	0.7614	228	tumor type=no tumor (normal/no finding/results negative), pancreas IPMT
6	6.1e-06	(2%)	0.75572	206	smoking=pooled, unknown, not applicable
7	1.2306e-06	(0.4%)	0.70936	6	Diabetes mellitus=manifest less than 1 year
8	1.1101e-06	(0.4%)	0.67289	4	WHO stage=I
9	6.5597e-06	(2.1%)	0.63842	218	alcohol consumption=pooled, unknown, not applicable
10	1.8227e-06	(0.6%)	0.63259	174	label=Cy5
11	7.544e-06	(2.4%)	0.62693	194	tumor site=no tumor(no finding/results negative),tumor subregion=no tumor(no finding/results negative)
12	3.6724e-06	(1.2%)	0.59997	264	pain=no
13	2.0443e-06	(0.7%)	0.57358	220	live status=alive without disease, died of other
14	6.3936e-06	(2.1%)	0.55894	204	pT stage=0
15	2.1795e-06	(0.7%)	0.55325	6	survival time(put 0 both if unknown or died in op)=6months
16	3.7573e-06	(1.2%)	0.53017	258	weight loss=no
17	4.1909e-06	(1.4%)	0.51555	268	weight loss in last 4 weeks=0kg
18	4.5148e-06	(1.5%)	0.5151	8	alcohol consumption=past,pre op treatment=chemotherapy
19	6.3414e-06	(2%)	0.49969	12	post op treatment=chemotherapy
20	3.7662e-06	(1.2%)	0.479	24	resection status=no tumor resection(e.g. cell line or biopsy of normal tissue)
21	3.045e-06	(1%)	0.47858	10	pancreatitis in history=unknown
22	7.544e-06	(2.4%)	0.45416	48	percentage normal=no tumor(no finding/results negative),normal=pancreas
23	7.5601e-06	(2.4%)	0.44925	16	tumor site=kidney
24	3.5021e-06	(1.1%)	0.44657	8	pN stage=1,WHO stage=III
25	2.1976e-06	(0.7%)	0.37825	4	tumor size=5cm,jaundice=yes
26	7.3106e-07	(0.2%)	0.33258	276	WHO stage=0
27	9.5421e-07	(0.3%)	0.33099	326	survival time(put 0 both if unknown or died in op)=0months
28	3.7708e-08	(0%)	0.32823	344	jaundice=none(no finding/results negative)
29	6.5841e-07	(0.2%)	0.32669	306	Diabetes mellitus=none(no finding/results negative)
30	1.3143e-07	(0%)	0.31356	338	pancreatitis in history=none(no finding/results negative)
31	3.2296e-06	(1%)	0.27898	258	pN stage=0
32	7.488e-06	(2.4%)	0.26798	22	tumor type=pancreas ductal adenocarcinoma, pancreas other
33	6.7822e-06	(2.2%)	0.26013	30	OP procedure=Whipple

34	1.8793e-06	(0.6%)	0.25592	266	pM stage=0
35	8.2917e-06	(2.7%)	0.24626	52	weight loss in last 4 weeks=4kg, 5kg, 6kg, 7kg
36	1.7092e-06	(0.6%)	0.22217	10	tumor size=4cm
37	9.7752e-07	(0.3%)	0.195	288	tumor size=0cm
38	5.7591e-06	(1.9%)	0.182	42	alcohol consumption=present
39	5.0081e-06	(1.6%)	0.15063	28	sex=male
40	1.6524e-06	(0.5%)	0.14178	20	OP date day=19,OP date day month=3,OP date day year=1997
41	6.6251e-07	(0.2%)	0.14126	12	CCD camera exposure time=70s
42	6.1085e-06	(2%)	0.12443	80	weight loss=yes
43	8.3137e-07	(0.3%)	0.089596	8	PMT setting=0%,laser power setting=0%
44	2.5492e-06	(0.8%)	0.087551	140	array series=7
45	7.8542e-06	(2.5%)	0.070192	62	tumor type=pancreas mucinous cystadenoma, pancreas mucinous cystadenocarcinoma
46	5.9167e-06	(1.9%)	0.052711	16	survival time(put 0 both if unknown or died in op)=12months
47	6.0069e-06	(1.9%)	0.051681	62	live status=alive with disease, died of disease
48	1.7103e-06	(0.6%)	0.049816	22	tumor size=3cm
49	2.8678e-06	(0.9%)	0.035576	16	metastasis location=pancreas
50	3.305e-08	(0%)	-0.020111	340	PMT setting=70%,laser power setting=70%
51	2.8824e-06	(0.9%)	-0.02669	10	OP procedure=pp Whipple,weight loss=unknown
52	4.4427e-06	(1.4%)	-0.028773	84	pain=moderate
53	2.8393e-06	(0.9%)	-0.031651	80	smoking=never
54	4.0245e-06	(1.3%)	-0.032038	24	tumor size=2cm
55	5.1321e-06	(1.7%)	-0.032907	102	pT stage=X (also for cell line of undescribed origin), 3
56	4.1636e-06	(1.3%)	-0.0411	60	array series=8
57	2.6611e-08	(0%)	-0.054731	336	CCD camera exposure time=0s
58	4.7678e-07	(0.2%)	-0.067383	304	OP date day=0,OP date day month=0,OP date day year=0
59	3.8013e-06	(1.2%)	-0.08853	68	tumor subregion=pancreas body, pancreas tail
60	1.4548e-06	(0.5%)	-0.11092	28	weight loss in last 4 weeks=8kg
61	1.3424e-06	(0.4%)	-0.1193	20	smoking=present
62	1.8808e-06	(0.6%)	-0.12992	146	race=caucasian,tumor or normal=tumor,tumor=pancreas carcinoma,differentiation grade (of tumor biopsy or original tumor in case of cell line)=X (also for cell line of undescribed origin),stroma=0
63	2.3433e-06	(0.8%)	-0.14103	16	WHO stage=not applicable
64	1.1446e-06	(0.4%)	-0.14412	42	pT stage=1, 2
65	3.3685e-06	(1.1%)	-0.15067	24	previous malignancy=yes
66	1.0526e-05	(3.4%)	-0.15225	36	tumor type=pancreas serous cystadenoma
67	3.8604e-06	(1.2%)	-0.16063	42	smoking=past
68	3.0505e-06	(1%)	-0.16155	82	pN stage=X (also for cell line of undescribed origin),pM stage=X (also for cell line of undescribed origin)
69	2.1985e-06	(0.7%)	-0.16567	64	array series=11
70	1.1193e-06	(0.4%)	-0.16812	44	WHO stage=II
71	1.2033e-06	(0.4%)	-0.16851	86	tumor subregion=unknown (also for cell line of undescribed origin), pancreas head
72	1.1512e-06	(0.4%)	-0.17808	134	birth date=specified

73	1.7965e-06	(0.6%)	-0.1793	134	post op treatment=no therapy
74	1.6817e-06	(0.5%)	-0.18911	138	pre op treatment=no therapy
75	2.2436e-06	(0.7%)	-0.1919	36	Diabetes mellitus=latent less than 1 year
76	1.9203e-06	(0.6%)	-0.19527	130	tumor site=pancreas
77	1.9675e-06	(0.6%)	-0.19686	94	OP procedure=pancreatic left resection, total pancreatectomy
78	1.3383e-06	(0.4%)	-0.20799	102	sex=female
79	2.1164e-06	(0.7%)	-0.21534	28	array series=12
80	9.5351e-07	(0.3%)	-0.21966	162	metastasis location=not applicable
81	8.2061e-07	(0.3%)	-0.24383	80	alcohol consumption=never
82	4.8657e-07	(0.2%)	-0.25169	174	label=Cy3
83	4.771e-06	(1.5%)	-0.27557	24	OP date day=7,OP date day month=5,OP date day year=2003
84	1.6575e-06	(0.5%)	-0.27827	8	tumor site=other
85	3.9765e-06	(1.3%)	-0.27975	56	array series=13
86	1.0839e-06	(0.3%)	-0.28622	300	percentage normal=<5%,normal=not applicable (tumor or cell line)
87	2.0671e-07	(0.1%)	-0.30447	324	resection status=X (also for cell line of undescribed origin)
88	9.8322e-07	(0.3%)	-0.30549	292	previous malignancy=none(no finding/results negative)
89	6.4435e-06	(2.1%)	-0.33191	66	live status=unknown
90	2.5777e-06	(0.8%)	-0.40528	62	birth date=unknown
91	5.3985e-06	(1.7%)	-0.46716	32	post op treatment=unknown,pre op treatment=unknown,previous malignancy=unknown
92	7.9986e-06	(2.6%)	-0.49382	50	race=unknown (also for cell line of undescribed origin)
93	3.7423e-06	(1.2%)	-0.51437	66	sex=unknown (also for cell line of undescribed origin)

Table 6: Cancer annotations after balancing the array production batches

Rank	χ^2 statistic	Inertia	Silhouettes	Values	Annotation
1	38.2398	0.000717878	0.103367	4	CA_19_9
2	36.5287	0.000685755	0.14347	4	tumor_type
3	35.6012	0.000668343	0.148896	4	live_status
4	34.7619	0.000652587	0.200492	3	smoking
5	32.1025	0.000602662	0.142022	3	weight_loss
6	31.3022	0.000587637	0.145338	4	tumor_subregion
7	31.3022	0.000587637	0.145338	4	OP_procedure
8	30.8476	0.000579104	0.120522	4	birth_date_day
9	30.8476	0.000579104	0.120522	4	birth_date_year
10	26.9903	0.00050669	0.0888086	3	pT_stage
11	25.4759	0.000478261	0.228657	3	sex
12	25.4759	0.000478261	0.228657	3	birth_date
13	21.1262	0.000396603	0.233851	3	metastasis_location
14	19.1818	0.0003601	0.14131	3	birth_date_month
15	19.1575	0.000359644	0.219194	2	Diabetes_mellitus
16	19.1575	0.000359644	0.219194	2	pain
17	19.1575	0.000359644	0.219194	2	weight_loss_in_last_4_weeks
18	16.9309	0.000317845	0.222145	3	alcohol_consumption
19	15.0322	0.000211044	-0.059447	3	array_series
20	10.1313	0.000190196	0.17127	2	WHO_stage
21	9.68265	0.000181773	0.142567	2	tumor_size
22	8.76135	0.000164477	0.397517	2	race
23	8.76135	0.000164477	0.397517	2	pre_op_treatment
24	8.76135	0.000164477	0.397517	2	post_op_treatment
25	8.76135	0.000164477	0.397517	2	tumor_or_normal
26	8.76135	0.000164477	0.397517	2	tumor
27	8.76135	0.000164477	0.397517	2	tumor_site
28	8.76135	0.000164477	0.397517	2	differentiation_grade
29	8.76135	0.000164477	0.397517	2	stroma
30	6.44896	9.88601e-05	0.146836	2	label
